# Global burden of latent multidrug-resistant tuberculosis: trends and estimates based on mathematical modelling

**DOI:** 10.1016/S1473-3099(19)30307-X

**Published:** 2019-08

**Authors:** Gwenan M Knight, C Finn McQuaid, Peter J Dodd, Rein M G J Houben

**Affiliations:** a Department of Infectious Disease Epidemiology, London School of Hygiene & Tropical Medicine, London, UK; b Centre for Mathematical Modelling of Infectious Diseases, London School of Hygiene & Tropical Medicine, London, UK; c TB Modelling Group, TB Centre, London School of Hygiene & Tropical Medicine, London, UK; d School of Health and Related Research, University of Sheffield, Sheffield, UK

## Abstract

**Background:**

To end the global tuberculosis epidemic, latent tuberculosis infection needs to be addressed. All standard treatments for latent tuberculosis contain drugs to which multidrug-resistant (MDR) *Mycobacterium tuberculosis* is resistant. We aimed to estimate the global burden of multidrug-resistant latent tuberculosis infection to inform tuberculosis elimination policy.

**Methods:**

By fitting a flexible statistical model to tuberculosis drug resistance surveillance and survey data collated by WHO, we estimated national trends in the proportion of new tuberculosis cases that were caused by MDR strains. We used these data as a proxy for the proportion of new infections caused by MDR *M tuberculosis* and multiplied trends in annual risk of infection from previous estimates of the burden of latent tuberculosis to generate trends in the annual risk of infection with MDR *M tuberculosis*. These estimates were used in a cohort model to estimate changes in the global and national prevalence of latent infection with MDR *M tuberculosis*. We also estimated recent infection levels (ie, in 2013 and 2014) and made predictions for the future burden of MDR tuberculosis in 2035 and 2050.

**Findings:**

19·1 million (95% uncertainty interval [UI] 16·4 million–21·7 million) people were latently infected with MDR tuberculosis in 2014—a global prevalence of 0·3% (95% UI 0·2–0·3). MDR strains accounted for 1·2% (95% UI 1·0–1·4) of the total latent tuberculosis burden overall, but for 2·9% (95% UI 2·6–3·1) of the burden among children younger than 15 years (risk ratio for those younger than 15 years *vs* those aged 15 years or older 2·65 [95% UI 2·11–3·25]). Recent latent infection with MDR *M tuberculosis* meant that 1·9 million (95% UI 1·7 million–2·3 million) people globally were at high risk of active MDR tuberculosis in 2015.

**Interpretation:**

We estimate that three in every 1000 people globally carry latent MDR tuberculosis infection, and prevalence is around ten times higher among those younger than 15 years. If current trends continue, the proportion of latent tuberculosis caused by MDR strains will increase, which will pose serious challenges for management of latent tuberculosis—a cornerstone of tuberculosis elimination strategies.

**Funding:**

UK Medical Research Council, Bill & Melinda Gates Foundation, and European Research Council.

## Introduction

Tuberculosis is the infectious disease responsible for the most deaths worldwide. The complex natural history of *Mycobacterium tuberculosis* means that, for ultimate disease control, people with latent infections need to be targeted.^1^, ^2^ Latent tuberculosis infection is defined as “a state of persistent immune response to stimulation by *M tuberculosis* antigens with no evidence of clinically manifest active tuberculosis”.^3^ As much as 23% of the world's population could have latent tuberculosis infection, and, even if transmission stopped in 2014, reactivation disease would overwhelm the 2035 End TB targets.^4^ Thus, understanding and targeting of latent tuberculosis is a priority for elimination,^2^ as recognised by the 2018 UN High-Level Meeting on Tuberculosis.^5^

Antimicrobial resistance is an increasingly serious threat to global public health.^6^ Multidrug-resistant (MDR) strains of *M tuberculosis*, which are resistant to both key first-line tuberculosis drugs (ie, rifampicin and isoniazid), are responsible for approximately a quarter of all deaths caused by antimicrobial-resistant infections.^7^ In 2017, MDR tuberculosis contributed to as estimated 14% of tuberculosis deaths globally.^8^ In patients with MDR tuberculosis, appropriate diagnosis is infrequent, treatment success is low, and treatment regimens are unacceptably long (ie, >18 months). MDR tuberculosis already accounts for a disproportionally large proportion of the financial burden for tuberculosis control programmes.^8^ Prevention of an increase in the incidence of MDR tuberculosis from a growing reservoir of latent MDR infection (ie, latent tuberculosis caused by MDR *M tuberculosis* strains) is therefore crucial for the success of any tuberculosis control programme.

Worryingly, MDR *M tuberculosis* strains are resistant to all recommended therapies for people with latent infection who are not known contacts of a person with active MDR tuberculosis disease.^3^ The priority population for testing and preventive therapy for latent infection is household contacts of tuberculosis patients,^9^ who are likely to be infected by their household member.^10^ However, in settings where the incidence of new infections is high, infection also frequently occurs outside the home.^10^ Because of such external transmission^11^ and the low proportion of people with active MDR tuberculosis disease detected (<30%),^8^ a substantial proportion of people with latent MDR infections will not have had recognised contacts with active MDR tuberculosis, and standard preventive therapy could be less effective. All diagnostics for latent tuberculosis infection rely on measurement of immune responses,^9^ and cannot establish the strain or susceptibility of *M tuberculosis*. Hence, estimation of the prevalence of latent MDR tuberculosis infection can help to inform estimates of the efficacy of standard therapy for latent infection. These estimates can also help to guide the use of tailored preventive treatment for contacts of patients with active MDR tuberculosis disease^3^, ^12^ and clarify the specific demand for new regimens (that include levofloxacin and delamanid) being tested.^13^, ^14^, ^15^

**Research in context**

**Evidence before this study**

We searched PubMed with the terms “(TB OR tubercul*) AND (global OR latent OR LTBI) AND burden AND model* AND (“resist*” OR “multidrug*” OR “MDR-” OR drug-resistant)” for articles published in English up to Nov 1, 2018. We identified 68 articles, including one by Houben and Dodd (our source for historical annual risks of infection), who estimated total global latent tuberculosis infections and the number of recent latent tuberculosis infections in each country that were resistant to isoniazid. Mehra and colleagues' mathematical modelling study showed that the burden of multidrug-resistant (MDR) latent tuberculosis infection in China was increasing, by contrast with the burden of drug-susceptible disease. Dodd and colleagues used a constant annual risk of infection to estimate the burden of MDR latent tuberculosis in children in a modelling study. By reviewing the reference lists of the results of our search, we identified a mathematical modelling study by Mills and colleagues exploring isoniazid preventive therapy, which showed that the burden of latent isoniazid-resistant infection was increasing under all scenarios (with and without preventive therapy) in Lesotho. However, we identified no studies in which the global burden of MDR latent tuberculosis infection was estimated in all age groups or in which historical trends in drug resistance were accounted for in estimations of the burden of MDR latent tuberculosis infection.

**Added value of this study**

In this study, we provide the first estimates of the global burden of latent MDR tuberculosis infection in all age groups across multiple settings. In the absence of empirical estimates, we have provided a robust modelling approach that accounts for the uncertainty in data for MDR tuberculosis to give 138 country-level estimates for the burden of latent MDR infection. We showed that the prevalence of latent MDR tuberculosis is higher in people younger than 15 years than in those aged 15 years or older. We estimate that, in 2015, approximately 2 million people were at increased risk of MDR tuberculosis disease after recent infection (ie, infection in 2013 or 2014).

**Implications of all the available evidence**

Targeting latent tuberculosis infection is essential for tuberculosis elimination, but standard preventive treatment regimens are probably ineffective against latent MDR strains. The estimates for the proportion of latent tuberculosis infections caused by MDR strains, and associated variations by setting and age we have provided should help to inform clinical decision making about regional preventive treatment regimens for latent MDR tuberculosis. Our estimates signal a worrying trend of an increasing burden of latent MDR tuberculosis in children younger than 15 years.

Additionally, as the overall incidence of tuberculosis and the annual risk of infection decrease, fewer people will be infected with latent tuberculosis (both drug-susceptible and MDR strains), which is thought to partly protect against reinfection with *M tuberculosis*.^16^, ^17^, ^18^ If the tuberculosis epidemic becomes increasingly driven by transmission of MDR strains, the presence of an existing protective primary drug-susceptible latent infection becomes less likely, which could further facilitate an increasing burden of MDR tuberculosis disease in younger generations.^19^ Thus, a decrease in the total prevalence of latent tuberculosis infection could result in MDR strains accounting for an increased proportion of latent infections overall.

No direct data for the prevalence of latent MDR tuberculosis are available, because infecting *M tuberculosis* strains cannot be isolated, and thus cannot be tested for resistance. Hence, a modelling approach is the only way to estimate this metric of the tuberculosis burden. We developed a new mathematical model that follows cohorts over time and applies historical annual risk of infection data to estimate trends in the risk of new infections with MDR *M tuberculosis* and the global prevalence of latent MDR tuberculosis infection.

## Methods

### Data sources

In this mathematical modelling study, we created a model that combined historical annual risks of infection (estimated in a previous study^4^) with trends in the proportion of new tuberculosis Cases that are MDR as a proxy for the proportion of the annual risk of infection that is MDR.^20^ This approach enabled us to estimate trends in the risk of MDR *M tuberculosis* infection, and hence the proportion of each cohort with latent tuberculosis infection that carried MDR *M tuberculosis*, and in turn to generate global estimates of the prevalence of latent MDR tuberculosis. As a starting point, we used Houben and Dodd's estimates^4^ of historical annual risks of infection with latent tuberculosis globally. They combined tuberculin skin test surveys with prevalence data and a revised Styblo rule to generate the annual risk of infection with any *M tuberculosis* strain for 168 countries.^4^ A previous systematic review showed that the prevalence of MDR tuberculosis in children and treatment-naive adults with tuberculosis was a reflection of the local transmission of MDR disease.^20^ Hence, we used the proportion of new tuberculosis cases (survey or surveillance data) reported to WHO's Drug Resistance Surveillance (DRS) project that were MDR as a proxy for the proportion of the annual risk of infection with *M tuberculosis* that was caused by MDR strains. 138 countries had available data from both Houben and Dodd's study^4^ and the DRS project. These countries accounted for 93% of all incident tuberculosis cases and 96% of the MDR tuberculosis burden in 2016, and included 28 of the 30 countries with a high burden of MDR tuberculosis according to WHO.^8^ The two high-burden countries not included (Angola and DR Congo) had no DRS data available; each contributed less than 1·5% of the estimated global incident MDR tuberculosis burden in 2016.^8^ Full details of country selection are in the appendix (pp 3–4).

### Model generation

We fitted curves to the data points in the WHO DRS data to generate trends for the country level proportions of new tuberculosis cases that MDR strains accounted for. Posteriors of potential fits provided 200 samples. These samples were multiplied by the trend estimates for the total annual risk of infection with *M tuberculosis*^4^ to give the annual risk of infection with either drug-susceptible or MDR *M tuberculosis* strains.

Curves were fitted through a flexible statistical model using a Bayesian Markov chain Monte Carlo approach in the *RStan* package in R.^21^ Our model allows for increases, stabilisation, and also subsequent decreases in the proportion of new cases that are MDR over time. In the absence of extensive timeseries data, the model was fitted with informative priors, which reflected three data-based assumptions about the trend characteristics of multidrug resistance, to constrain the potential pattern of increases in the prevalence of MDR tuberculosis disease. First, we assumed that the appearance of detectable levels of MDR tuberculosis in any country before 1970 was very unlikely (appendix p 5). To capture this time constraint, the model fitted for each country a time when the proportion of new tuberculosis cases that were MDR was assumed to be measurable. The prior for this parameter was normally distributed with a mean of 1985 and a 95% range between 1970 and 2000, which matched a previous modelling study's assumption that transmissible MDR strains of *M tuberculosis* strains emerged 20–60 years before 2013.^22^ Second, the rate of increase in the proportion of new cases that were MDR was governed by two parameters (appendix pp 6–10) that were scaled to prevent too rapid an increase while still capturing the wide range of MDR prevalences (including some very low rates of increase). Third, we ensured that our model did not allow for a peak and crash in the proportion of new tuberculosis cases that were MDR. The details for the choice of priors and plots of the trends generated by these assumptions are in the appendix (pp 6–10, 19–27).

### Model outcomes

We inputted estimates of the annual risk of infection for both drug-susceptible and MDR *M tuberculosis* into a new cohort model, and tracked the proportion of individuals by age infected with drug-susceptible or MDR strains (appendix pp 14–16) from 1934 to 2014. The initial conditions were calculated assuming a constant annual risk of infection before 1934, and we assumed that there was no MDR tuberculosis before 1960. Estimates of the burden of latent tuberculosis infection in 2014 are presented, because 2014 was the final timepoint in the annual risk of infection trends.^4^ On the basis of previous work,^16^ our model assumed that 79% of people with latent tuberculosis infection were protected against reinfection. We included all available WHO DRS data in the fitting process—ie, for trends in the proportion of new tuberculosis cases that is MDR, we used WHO data up to 2018.

Specifically, the burden we aimed to characterise was the number of individuals with a persistent immune response to stimulation by *M tuberculosis* antigens without evidence of clinically manifested active disease.^3^ We report the resistance status (ie, drug susceptible or MDR) of the last infecting strain, taking into account protection against reinfection, ignoring dual infections, and, in the absence of a quantitative alternative, assuming lifelong infection.

We used model outputs to estimate the population infected with MDR *M tuberculosis* in 2013 or 2014 (ie, the most recent 2 years for which annual risk of infection trends^4^ were available), who would be at a higher risk of progressing to active MDR tuberculosis in 2015 than those infected earlier. We calculated the risk ratio (RR) of people younger than 15 years having latent MDR tuberculosis (conditional on being latently infected) compared with those aged 15 years or older. We chose 15 years as the age cutoff to match previous age-segregated estimates of latent tuberculosis infection and because it is a standard cutoff in the natural history of tuberculosis, as reflected in WHO data.^8^

To estimate the contribution of latent MDR tuberculosis infections in 2015 to disease burden in 2035 and 2050, we assumed no transmission of *M tuberculosis* after 2014. We used UN Population Division demographic projections^23^ to estimate the burden of latent MDR tuberculosis in 2035 and 2050 and the incidence of active MDR disease assuming a 0·03% per year remote activation rate, and explored this value in a sensitivity analysis.^24^ We compared our results to the overall WHO End TB targets of less than ten tuberculosis cases per 100 000 population by 2035, and the Stop TB target of less than one case per million population by 2050.^1^

To compare how well our estimates of the burden of latent MDR tuberculosis were informed by resistance data, we used the cohort model to establish the proportion of latent infection in 2014 in each country in each 5-year time block in the past. The sum of all 5-year time blocks for which any WHO DRS data were available gave the data coverage value (appendix pp 28–32). This metric combined the contribution of a specific period to the burden of latent MDR tuberculosis with whether data were present during that time to compare the availability of data for MDR infection by setting. The higher the value, the greater the overlap between contribution of a period to the burden of latent MDR infection and data availability.

### Sensitivity analysis

The fitness costs associated with the appearance of resistance within *M tuberculosis* strains are debated.^25^, ^26^ We did a sensitivity analysis in which we applied a 40% fitness cost^25^ to the protection from reinfection (ie, a reduction from 79% to 47%), and separately to the rate of reactivation (ie, a reduction from 0·03% per year to 0·018% per year), in people with latent MDR tuberculosis. We also did a sensitivity analysis on trend shape, in which we allowed for more flexible dynamics in the annual risk of infection with MDR tuberculosis in a subset of countries with sufficient data and a potential peak and crash in the proportion of all cases of disease that were caused by MDR strains (appendix pp 11–14).

### Role of the funding source

The funder of the study had no role in study design, data collection, data analysis, data interpretation, or writing of the report. The corresponding author had full access to all the data in the study and had final responsibility for the decision to submit for publication.

## Results

**Figure 1:**
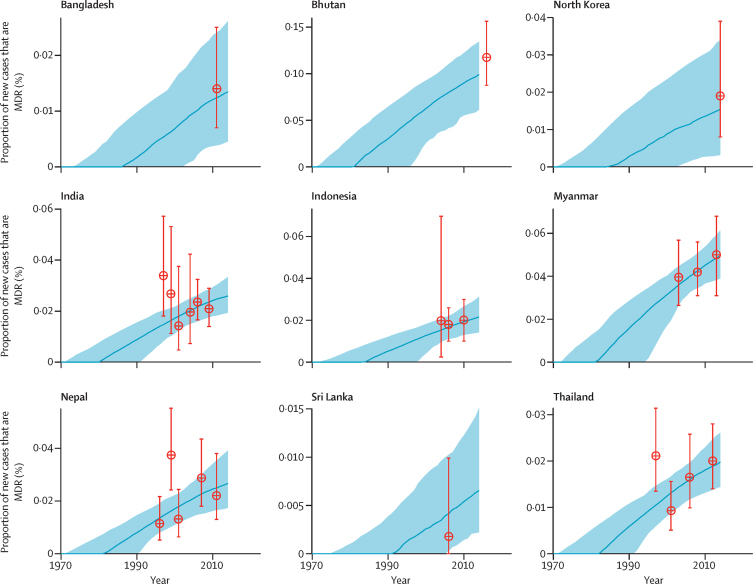
Proportion of new cases of tuberculosis disease accounted for by MDR tuberculosis in the nine countries in the WHO South-East Asia region included in our model The blue lines represent the median proportion from 200 model fits to WHO data (the red datapoints; error bars show 95% CIs). The shaded regions represent the 95% uncertainty intervals. Although we estimated the burden of latent MDR tuberculosis in 2014 (hence the cutoff in this figure) the model trend was fitted to all WHO Drug Resistance Surveillance data. MDR=multidrug resistant.

Estimates of the proportion of new cases of tuberculosis that MDR tuberculosis accounted for were provided by model fits for all 138 countries, and closely matched WHO data (appendix pp 19–27). Examples of model fits for countries in the WHO South-East Asia region are shown in figure 1, which shows the substantial uncertainty associated with the lack of data before 1990 and the rising trend in the proportion of new cases that are caused by MDR tuberculosis across all countries.

We estimated a global prevalence of latent MDR tuberculosis infection in 2014 of 0·3% (95% uncertainty interval [UI] 0·2–0·3; table 1), representing 19·1 million (95% UI 16·4 million–21·7 million) people (table 2). 1·2% (95% UI 1·0–1·4) of the global burden of latent tuberculosis infection was due to MDR strains. Our estimates of the overall prevalence of latent tuberculosis infection (ie, both drug-susceptible and MDR infections; table 1) matched those from previous modelling,^4^ validating the cohort model approach that we used.

**Table 1:** Prevalence of latent tuberculosis infection, 2014, by WHO region

	**Prevalence (95% uncertainty interval)**		**Proportion (95% uncertainty interval)**	
	Drug-susceptible latent tuberculosis	Multidrug-resistant latent tuberculosis	Latent tuberculosis that is multidrug resistant	Latent tuberculosis that is multidrug resistant in people younger than 15 years
African	22·1% (20·1–25·5)	0·23% (0·19–0·29)	1·0% (0·8–1·3)	2·3% (1·9–2·7)
Americas	10·6% (7·3–19·0)	0·05% (0·04–0·06)	0·5% (0·3–0·8)	3·3% (2·8–4·1)
South-East Asia	30·7% (27·7–34·5)	0·31% (0·23–0·41)	1·0% (0·7–1·3)	2·2% (1·9–2·6)
Eastern Mediterranean	16·4% (13·5–20·9)	0·14% (0·08–0·24)	0·9% (0·5–1·5)	2·9% (1·9–3·8)
Western Pacific	26·8% (17·8–39·2)	0·36% (0·26–0·49)	1·3% (0·7–2·2)	3·7% (3·3–4·1)
European	13·5% (9·9–19·8)	0·38% (0·32–0·44)	2·8% (1·6–3·9)	14·1% (13·1–15·2)
Global	22·9% (20·1–26·1)	0·28% (0·24–0·31)	1·2% (1·0–1·4)	2·9% (2·6–3·1)

**Table 2:** Number of people with latent tuberculosis infection, 2014, by WHO region

	**Drug-susceptible latent tuberculosis (thousands)**	**Multidrug-resistant latent tuberculosis (thousands)**
African	155 000 (141 000–179 000)	1590 (1310–2010)
Americas	102 000 (70 700–183 000)	510 (418–624)
South-East Asia	584 000 (527 000–656 000)	5810 (4410–7750)
Eastern Mediterranean	96 000 (78 900–122 000)	837 (481–1410)
Western Pacific	493 000 (326 000–720 000)	6620 (4840–9000)
European	122 000 (90 100–180 000)	3440 (2920–3990)
Global	1 580 000 (1 380 000–1 800 000)	19 100 (16 400–21 700)

Data are n (95% uncertainty interval).

**Figure 2:**
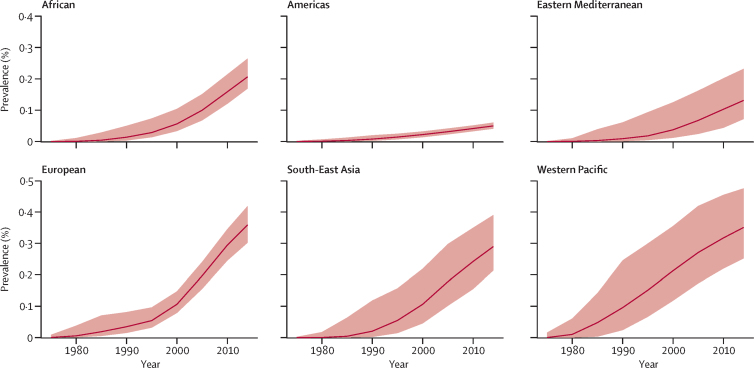
Prevalence of latent multidrug-resistant tuberculosis infection, by WHO region The red line represents the median from 200 model fits. The shaded region represents the 95% uncertainty interval.

**Figure 3:**
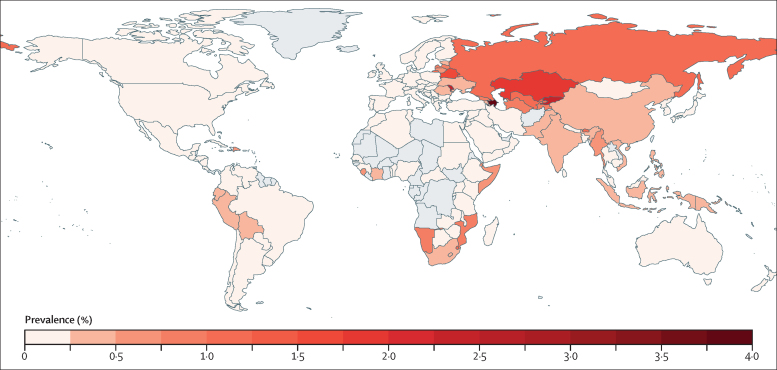
Estimated worldwide prevalence of latent multidrug-resistant tuberculosis infection Countries with no data are shown in grey. Equivalent maps for 2035 and 2050 estimates are in the appendix.

Our model's outputs reflected data showing that the proportion of tuberculosis disease caused by MDR strains is increasing, and suggested that the prevalence of latent MDR tuberculosis infection has increased substantially across all six WHO regions since 1990 (figure 2). Our estimates of the prevalence of latent MDR tuberculosis infection varied geographically, with the lowest prevalence in the WHO Americas region (0·1% [95% UI 0·0–0·1]) and the highest in the WHO European region (2·8% [1·6–3·9]; figure 2; table 1). Most countries included in our model had a prevalence of latent MDR tuberculosis of less than 1%, but prevalence in some countries in eastern Europe and central Asia was higher than 1·5% (figure 3; appendix p 17). Among the 30 countries with the largest burden of MDR tuberculosis, the proportion of latent tuberculosis caused by MDR was highest in Kazakhstan (17·5% [95% UI 6·5–22·9]). China (approximately 6 million), India (4 million), and Russia (1·8 million) had the highest absolute numbers of people with latent MDR tuberculosis (appendix p 17).

**Figure 4:**
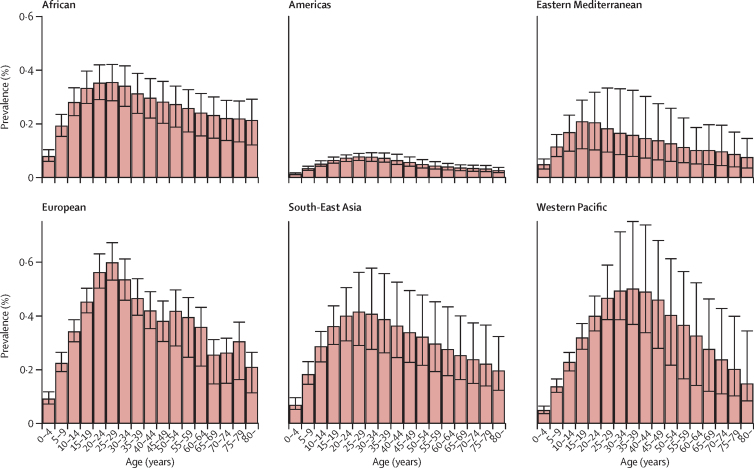
Prevalence of latent multidrug-resistant tuberculosis infection in each age group, by WHO region Error bars show 95% uncertainty intervals. The prevalence of latent infection with drug-susceptible tuberculosis is shown in the appendix.

In children younger than 15 years, the global proportion of latent tuberculosis that was caused by MDR strains was 2·9% (95% UI 2·6–3·1)—more than double that in the overall population (table 1). In the WHO European region, 14·1% (95% UI 13·1–15·2) of latent tuberculosis in children younger than 15 years was caused by MDR strains, compared with 2·8% (1·6–3·9) in the total population (table 1). In most WHO regions there was a peak in latent MDR tuberculosis infections between ages 20 years and 35 years (figure 4). The RR for a latent infection being caused by an MDR strain in people younger than 15 years versus those aged 15 years or older was 2·65 (95% UI 2·11–3·25).

The number of people infected with latent MDR tuberculosis infection in 2013 and 2014 was estimated to be 1·9 million (95% UI 1·7 million–2·3 million)—0·03% (95% UI 0·02–0·03) of the global population in 2014. We estimated that 0·6 million (95% UI 0·6 million–0·8 million) children younger than 15 years had been infected with latent MDR tuberculosis in 2013 and 2014, who were therefore at high risk of progression to MDR tuberculosis disease in 2015.

Assuming no ongoing transmission from 2015, we projected 14 million (95% UI 12 million–16 million) cases of latent MDR tuberculosis surviving until 2035, decreasing to 11 million (95% UI 10 million–12 million) by 2050 (appendix). Incidence of MDR disease from this latent pool would be 0·5 (95% UI 0·4–0·5) per million people per year in 2035 and 0·3 (0·3–0·4) per million people per year in 2050. These estimates do not exceed the 2035 WHO End TB or 2050 Stop TB targets.^1^

The mean of the metric for data coverage across all countries and model fits was 0·56 (range 0–1). 14 countries had median metric values of 1, suggesting that data were available within all contributing periods (appendix pp 29–30). Four countries (Bhutan, Djibouti, Sudan, and Togo) had no metric values because data were available only after 2014 (appendix pp 29–30). The best data coverage for the 30 countries with the highest burden of MDR tuberculosis was in Russia, Thailand, and Uzbekistan (median metric values 0·87–0·99); Zimbabwe was the only of these countries with a median metric value less than 0·25 (appendix pp 29–30). The top four countries in terms of absolute numbers of people infected with latent MDR tuberculosis (appendix p 18)—ie, China, India, Indonesia, and Russia—had median metric values higher than 0·5.

In sensitivity analyses, a 40% reduction in the protective effect of latent MDR tuberculosis against reinfection resulted in a less than 1% difference to all our results for the prevalence of latent MDR tuberculosis (appendix p 33). The RR for latent MDR tuberculosis infect by age decreased slightly to 2·47 (95% UI 2·03–2·97). A 40% fitness cost affecting progression from latent MDR disease to active MDR disease (reactivation) reduced the incidence of active disease to 0·29 (95% UI 0·25–0·32) per million per year in 2035 and to 0·20 (0·18–0·23) per million per year in 2050. Allowing for more flexible dynamics resulted in a pre-1995 peak (ie, before data were available) in the proportion of new tuberculosis cases that MDR strains account for in the three countries included (ie, China, India, and the USA). This peak increased estimates of the overall prevalence of latent MDR tuberculosis infection but had little effect on estimates in people younger than 15 years (appendix pp 36–38).

## Discussion

We estimated that, in 2014, the global prevalence of latent MDR tuberculosis infection was 0·3% (95% UI 0·2–0·3). Prevalence varied substantially by WHO region and age group, but was increasing in all regions. The proportion of latent infections with MDR tuberculosis strains was 1·2% (95% UI 1·0–1·4) in the overall population, but more than double that in children younger than 15 years. We estimated that if all transmission stopped in 2015, the number of new cases caused by reactivation of latent MDR tuberculosis infection would not exceed the one per million targets for tuberculosis elimination by 2050, but would contribute approximately a third of the target.^1^

Our analysis suggests that prevalence of latent MDR tuberculosis peaks in people aged 20–35 years, reflecting the combination of lower prevalence of latent infections in younger age groups because of lower cumulative exposure time and an increase in the proportion of infections caused by MDR strains beginning in the early 1990s. A high burden of latent MDR tuberculosis in children was estimated previously, but the model on which this estimate was based assumed a constant annual risk of infection over time and did not consider other age groups.^27^ We showed that children had double the risk of having a latent tuberculosis infection that was caused by a MDR strain compared with adults, which is worrying in view of the higher frequency of progression to active disease and lower probability of appropriate diagnosis or treatment for MDR tuberculosis in children compared with adults.^28^ In terms of the future burden of MDR tuberculosis, these children also represent a long-persistent reservoir for MDR disease in the absence of substantial and effective rollout of new preventive therapy programmes for latent tuberculosis. This burden is driven by the increasing proportion of the annual risk of infection that is accounted for by MDR tuberculosis as the overall annual risk of infection decreases globally. Hence, children are more likely to be infected with MDR *M tuberculosis* than the current generation of adults were. Indeed, the current generation of adults are partly protected by the higher prevalence of latent drug-susceptible tuberculosis infection,^16^, ^17^, ^18^ which makes them less likely to have latent MDR tuberculosis infection. The public health implications of these findings are that, independently of contact with people with active MDR tuberculosis, latent tuberculosis infections in children should be considered more likely to have been caused by an MDR strain.

The WHO regions with the highest prevalence of latent MDR tuberculosis infection (ie, the European and Western Pacific regions) in 2014 in our model were the regions with the highest proportion of new cases of tuberculosis caused by MDR strains in the WHO 2018 Global TB report.^8^ These estimates, alongside country-level estimates, should help to guide preventive therapy in some settings: a high proportion of latent tuberculosis infections being caused by MDR *M tuberculosis* in high incidence settings could suggest that standard preventive therapy for latent infections should be given with even more caution to household contacts and that possible second-line therapies, such as those being trialed,^13^, ^14^, ^15^ should be considered.

Our estimates showed that the prevalence of latent MDR tuberculosis is increasing in all regions, as has been estimated previously for China^29^ and for latent isoniazid-resistant infection in Lesotho,^30^ despite WHO estimates suggesting that the incidence of new cases of MDR tuberculosis has stabilised. Our aim was to characterise historical patterns of change in the annual risk of infection with MDR strains to inform the burden of latent MDR tuberculosis rather than to establish trends in the incidence of MDR disease.

We have created a generalisable approach that combines historical country-level data with generally informative priors on emergence of MDR tuberculosis disease to estimate the global prevalence of latent MDR tuberculosis infection. We used informative priors to capture the timings of isoniazid and rifampicin use and limited rates of increase to better support the data available. A strength of our approach is the inclusion of a range of trajectories for the proportion of new cases of tuberculosis that are caused by MDR strains. Our model could also track infection by age, which showed the increasing burden of latent MDR disease in younger age groups. We also included sensitivity analyses around the effect of MDR disease on protection from reinfection and the rate of reactivation, which showed that the former had little effect on our results whereas the latter reduced predicted incidence of active MDR tuberculosis by approximately 40% (in line with the assumed parameter reduction). This finding highlights the importance of establishing the rate of reactivation for forecast analysis.

However, our analysis also had several limitations. First, we relied on historical trends for the proportion of new cases of tuberculosis that was due to MDR strains, even though data for before 1990 are scarce. By setting relatively informative priors (eg, the emergence of MDR strains before 1970 is very unlikely) and allowing for both quadratic and linear curves, we think that we have explored a reasonable range of potential MDR trends and reflect this in our wide uncertainty ranges, but this analysis was fundamentally limited by the amount and precision of data. We explored an even wider range of potential trends in a sensitivity analysis for a small set of countries with a high burden of MDR disease, for which data suggested a potential peak in the annual risk of infection with MDR tuberculosis before data were available, by fitting more flexible spline models. Although this peak affected our results, it assumed that transmission of MDR tuberculosis rose rapidly from 1970 onwards and pushed the limits of the available data. However, the overall prevalence of latent MDR tuberculosis infection is clearly sensitive to data and assumptions for pre-2000 trends, and future work could include past trend determination, especially for China (possibly through phylogenetic analysis), because alternative potential past trends contributed most to the estimated change in the prevalence of latent MDR tuberculosis. Our data availability metric shows that most of the 30 countries with the highest prevalence of MDR infection had good data availability (ie, median metric values higher than 0·5). New drug resistance surveys or improved surveillance are needed to estimate recent prevalence of MDR tuberculosis, which could then be used to update our estimates of the prevalence of latent MDR tuberculosis. When countries had both survey and surveillance data available, we did not treat the data differently (eg, to account for potential under-reporting in surveillance).

A second limitation was the homogeneity assumed in the model in terms of contact patterns, strain differences, reactivation rates, spatial variation, and population characteristics. By not including differences in mixing patterns by age, we might have missed some age variation: age-assortative mixing, combined with changing disease presentation,^31^ could result in further differences between children and adults. In terms of strain variation, reduced reactivation rates for MDR tuberculosis had a substantial effect on the future incidence of MDR disease in our model, suggesting that strain variation differences in reactivation rates could drive differences in MDR incidence globally. For example, future work could estimate variance in the prevalence of latent MDR tuberculosis by HIV status, which could have consequences for assumed fitness costs to resistance and hence could be associated with a greater prevalence of latent MDR tuberculosis infection in HIV-positive populations. Similarly, we modelled, and averaged surveillance and survey data, at the national level, which for some settings, such as Russia, might not be appropriate.

We also assumed lifelong infection, despite the likelihood that self-cure is possible after infection with *M tuberculosis*.^19^ Most estimates of the prevalence of latent tuberculosis infection are based on tuberculin skin tests. Some people with positive tuberculin skin test results could have cleared their *M tuberculosis* infection, suggesting that the reservoir of true infection for reactivation is smaller than the estimated prevalence of latent infection (which is defined through persistent immune response).^32^ We also present data for recent infections (ie, in 2013 and 2014), which would have caused most cases of active MDR tuberculosis disease in 2015. We accounted for our potential overestimation of the population carrying MDR *M tuberculosis* in the estimated future number of people with reactivation MDR disease that arises from the reservoir of latent MDR infection The implications for global elimination targets are likely to be robust. We also modelled only multidrug resistance and not all rifampicin resistance, because historical data were available only for the former.

A further complexity that we did not explore was mixed infection, because the dynamics of reactivation and mixed strain disease, although important, are not fully understood.^33^ Latent infection was assumed to be caused by the last successfully infecting strain (eg, either MDR or drug-susceptible *M tuberculosis*, taking into account protection against reinfection). However, in view of the relatively low annual risk of infection since the rise of MDR prevalence from around 1985, compared with the decades before, potential mixed infections probably account for a low proportion of all cases of latent MDR tuberculosis, and are unlikely to affect the total number of cases with latent MDR or drug-susceptible infection.

Furthermore, in our main analysis we used a single-study estimate for the level of protection against reinfection progression conferred by latent infection,^16^ despite the availability of other estimates.^17^, ^18^ This estimate used in our model captured potential risk reductions in infection and progressive tuberculosis dependent on latent tuberculosis status, which currently cannot be separated. We explored the effect of this parameter by lowering the protection against reinfection conferred by latent MDR tuberculosis infection in our sensitivity analysis, which showed a less than 1% change to the prevalence of latent MDR infection. We did not include a sensitivity analysis for all latent tuberculosis infections because the effect on MDR disease, and the effect in previous work^4^ of reducing protection from the mean 79% that we used to 50% for all latent infections, were so small. The only effect noted in previous work,^4^ which was reflected in the slightly reduced risk ratio for latent MDR tuberculosis by age in our sensitivity analysis, was on the age distribution of recent infections, with an increased proportion of infections in older age groups.

The broader public health implications of this work are that evidence for the efficacy of preventive therapies for people potentially infected with latent MDR tuberculosis needs to be strengthened and the recommendations possibly made context specific. Our estimates also provide some idea of the value of a diagnostic test to differentiate between the resistance status of latent tuberculosis strains. In terms of future modelling work, the rate and proportion of self-clearance of latent tuberculosis infection should be quantified to better estimate the size of the reactivation reservoir. For future transmission, the effect of any fitness costs conferred by resistance carriage in *M tubercolosis* on natural history progression should be explored, and trends in the annual risk of infection with MDR *M tuberculosis* should be further established.

Our estimates suggest that one in every 83 individuals with latent tuberculosis is infected with a MDR strain of *M tuberculosis*, which means that nearly three in every 1000 people globally carry latent MDR tuberculosis. However, among children younger than 15 years, one in every 34 people with latent tuberculosis is infected with MDR strains, and this number will be even higher among close contacts of people with active MDR disease. We also found that the prevalence of latent MDR tuberculosis infection is increasing in all WHO regions. Under current trends, the proportion of latent disease caused by MDR strains is only going to increase, with serious implications for control of latent tuberculosis infections—a cornerstone of tuberculosis elimination strategies.
