# Correction to *Lancet Infect Dis* 2019; 19: 903–12

**DOI:** 10.1016/S1473-3099(20)30087-6

**Published:** 2020-04

*Knight GM, McQuaid CF, Dodd PJ, Hoube RMGJ. Global burden of latent multidrug-resistant tuberculosis: trends and estimates based on mathematical modelling.* Lancet Infect Dis *2019; **19**: 903–12*—In the Summary of this Article, the first sentence of the Interpretation should have read “prevalence of MDR *M tuberculosis* in those with latent tuberculosis infection is more than double among those younger than 15 years”. This correction has been made to the online version as of Feb 17, 2020.

