# Supplementary material for: Global burden of latent multidrug-resistant tuberculosis: trends and estimates based on mathematical modelling
Source: Lancet Infect Dis. 2019 Aug;19(8):903–12. doi: 10.1016/S1473-3099(19)30307-X (PMC6656782; doi:10.1016/S1473-3099(19)30307-X)
Supplement: Supplementary appendix [file mmc1.pdf]

THE LANCET Infectious Diseases

Supplementary webappendix

This webappendix formed part of the original submission and has been peer reviewed. We post it as supplied by the authors.

Supplement to: Knight GM, McQuaid CF, Dodd PJ, Houben RMGJ. Global burden of latent multidrug-resistant tuberculosis: trends and estimates based on mathematical modelling. *Lancet Infect Dis* 2019; published online July 4. http://dx.doi.org/10.1016/S1473-3099(19)30307-X.

Appendix for ”The global burden of multi-drug resistant latent tuberculosis: recent trends and estimates using mathematical modelling”

Gwenan M. Knight, Finn McQuaid, Peter J. Dodd* & Rein M. G. J. Houben*

Contents

1 Additional Methods **3**
1.1 Details on countries included 3
1.1.1 Country selection 3
1.1.2 MDR-TB incidence case burden in 2016 4
1.1.3 Excluded high MDR-TB countries 4
1.1.4 Countries with only sub-national data 4
1.2 WHO data 5
1.2.1 When does WHO DRS data exist? 5
1.3 Priors for parameters in model for trend 6
1.3.1 Appearance of MDR-TB 6
1.3.2 Equation for trend 6
1.3.3 Rate of increase 7
1.3.4 Proposed priors 8
1.3.5 Effect of each parameter on trend 8
1.3.6 Model fitting details 9
1.4 Sensitivity analysis 3: trend analysis 11
1.4.1 Countries included in sensitivity analysis 3 11
1.4.2 Complex curve fitting in sensitivity analysis 3 11
1.5 Cohort model 14
1.5.1 Combining the data 14
1.5.2 Model construction 14
1.5.3 Initial conditions 15
1.5.4 Key assumptions 16

1.6 Metric for MDR-LTBI data coverage 17
1.7 Code 17

2 Additional Results **18**
2.1 Top 10 countries by number with MDR-LTBI 18
2.2 Model fits for each country 19
2.3 Proportion infected by age 28
2.4 Metric for MDR-LTBI data availability 29
2.5 Sensitivity analysis 1 33
2.6 Individual country estimates 34
2.7 Map of MDR-LTBI prevalence in 2035 and 2050 34
2.8 Sensitivity analysis 3: trend analysis 36
2.8.1 Spline curve fit 36
2.8.2 Estimated levels 36

1 Additional Methods

1.1 Details on countries included

1.1.1 Country selection

Previous work estimating annual risk of infection (ARI) with *Mycobacterium tuberculosis* included 168 countries: 24 had been dropped as they could not be matched across the TB and population data, 24 countries as they had too small a population size (<500,000) and 4 because they had fewer than 15 data points in total [1].

The WHO Drug Resistance Surveillance (DRS) Project has data on multi-drug resistant (MDR-) TB burden for 159 countries from surveys or surveillance. As in Houben & Dodd, for Serbia and Montenegro we used MDR burden estimates reported for the combined country from 1990 to 2004 for the two countries separately, and MDR burden estimates specific to each country thereafter.

Only 138 countries were included in both the original ARI estimates and in the WHO MDR data. 21 countries were in the WHO MDR data but not in the ARI estimates. 30 were in the ARI estimates but not in the WHO MDR data.

The 21 countries included in the MDR WHO data but not in the ARI estimates were excluded from the ARI estimates for the following reasons (ISO3 codes):
Excluded from ARI as data could not be matched (n=8): ”AND” ”BMU” ”CYM” ”COK” ”DMA” ”MHL” ”MNP” ”PLW”
Excluded from ARI as small population (n=12): ”BHS” ”BRB” ”BRN” ”CUW” ”PYF” ”GUM” ”ISL” ”MLT” ”NCL” ”WSM” ”SYC” ”VUT”
Excluded from ARI as < 15 data points (n=1): ”BES”

The 30 countries included in the ARI estimates but not in the MDR WHO data were (ISO3 codes): ”MLI” ”SSD” ”NER” ”COM” ”LBR” ”GNQ” ”GAB” ”GHA” ”BFA” ”AGO” ”MRT” ”GNB” ”CPV” ”COD” ”ERI” ”CMR” ”COG” ”BDI” ”TCD” ”GUY” ”HTI” ”SUR” ”PAN” ”TTO” ”LBY” ”AFG” ”PSE” ”ARE” ”SLB” ”LAO”.

These account for 4.7% of the 2014 global population, 6.8% of the number of incident MDR TB cases in 2016 and 6.5% of the 2014 incident TB burden. Each individual country contributes ¡ 1% of the total 2014 incident TB burden except the Congo (2.3%) (”e_inc_num” in WHO code book).

The final 138 (in terms of iso3 codes) were:
”ETH” ”NGA” ”KEN” ”ZMB” ”CIV” ”TGO” ”ZAF” ”ZWE” ”TZA” ”SWZ” ”LSO” ”RWA” ”MWI” ”CAF” ”MDG” ”UGA” ”DZA” ”SLE” ”NAM” ”SEN” ”BEN” ”BWA” ”GIN” ”GMB” ”MUS” ”MOZ” ”GTM” ”PRI” ”PRY” ”COL” ”JAM” ”ECU” ”MEX” ”CRI” ”ARG” ”VEN” ”URY” ”USA” ”NIC” ”BOL” ”BRA” ”DOM” ”SLV” ”CAN” ”CHL” ”HND” ”CUB” ”PER” ”EGY” ”SOM” ”JOR” ”OMN” ”SDN” ”SAU” ”LBN”

”DJI” ”MAR” ”KWT” ”PAK” ”IRN” ”IRQ” ”BHR” ”QAT” ”SYR” ”TUN” ”YEM” ”LVA” ”IRL” ”CZE” ”CYP” ”GRC” ”MNE” ”MDA” ”AUT” ”FRA” ”BLR” ”DEU” ”BIH” ”NLD” ”ARM” ”HRV” ”LUX” ”SWE” ”RUS” ”GBR” ”TJK” ”GEO” ”SVN” ”NOR” ”LTU” ”PRT” ”AZE” ”ESP” ”CHE” ”POL” ”TKM” ”BEL” ”TUR” ”SVK” ”BGR” ”EST” ”UKR” ”FIN” ”ALB” ”MKD” ”SRB” ”DNK” ”HUN” ”ITA” ”KGZ” ”UZB” ”ISR” ”KAZ” ”ROU” ”IND” ”THA” ”PRK” ”BTN” ”NPL” ”LKA” ”BGD” ”IDN” ”MMR” ”JPN” ”FJI” ”CHN” ”NZL” ”KOR” ”MYS” ”MAC” ”KHM” ”SGP” ”AUS” ”PHL” ”PNG” ”HKG” ”MNG” ”VNM”

1.1.2 MDR-TB incidence case burden in 2016

To calculate the number of MDR-TB incident cases in 2016 (for estimation of relation proportion of MDR-TB included), the average of the percentage of new and previously treated cases that was MDR-TB of those rifampicin resistant was calculated. This average was used to multiply the number of incident rifampicin resistant cases in each country. The levels in 2016 were used as this was the first year that estimates for all countries had been reported, either from survey or surveillance data or a modelled level.

1.1.3 Excluded high MDR-TB countries

Two of the 30 high MDR-TB burden countries [2] were missing from the 138: Angola and The Democratic Republic (DR) of the Congo. Both settings have had no drug resistance survey despite high levels of MDR-TB having been observed. In Angola, especially high levels are seen in retreatment cases (e.g. 71% [3]) whilst in the DR Congo civil unrest has left the country with fragile health systems and a scarcity of data on MDR levels [4]. Despite being in the top TB and TB-HIV burden countries, we excluded both countries as there was no WHO DRS data.

1.1.4 Countries with only sub-national data

Seven countries (ISO3: ”BRA” ”CAF” ”GIN” ”IDN” ”IND” ”PNG” ”PRK”), have only sub-national data. As these included three of the 30 high MDR settings (India, Indonesia and Papua New Guinea) it was decided that these could not be removed. Instead, where multiple sub-national surveys were taken on the same year a simple average was taken, and the overall percentage of new cases that was MDR from the sub-national surveys was used as an estimate for the whole country. This was also true for countries where multiple sub-national surveys were taken on single years from different sub-national regions (e.g. Oblasts for Russia).

1.2 WHO data

1.2.1 When does WHO DRS data exist?

The data provided by the WHO on the proportion of MDR in new and previously treated cases is mostly from the year 2000 onwards (Figure 1). Only one country (Algeria) has any data before 1990.

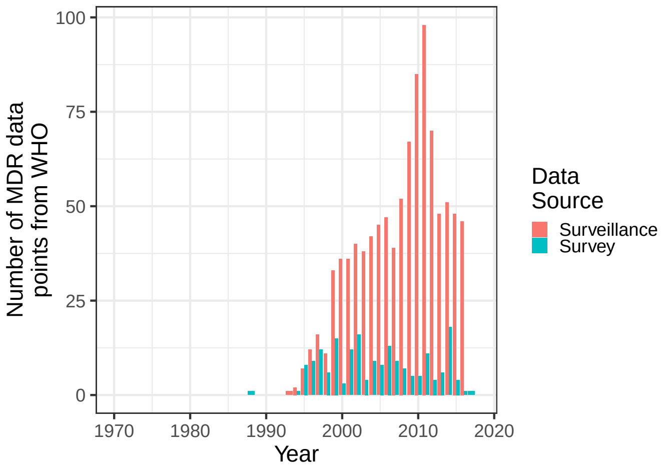

Figure 1: The number of data points (countries or regions) with data at each yearly time point from WHO DRS Project

The uncertainty in the WHO data is given for the data collected from surveys. For the data collected from surveillance a 95% Binomial Confidence interval was calculated (using the Wilson method) and used in the analysis.

1.3 Priors for parameters in model for trend

1.3.1 Appearance of MDR-TB

For each country a year in the past, t_m, when MDR-TB appeared in a country was fitted. We are interested in MDR-LTBI in 2014. In 1970, it was first shown that including rifampicin (or pyrazinamide) alongside isoniazid, within the then "standard" streptomycin / isoniazid regimen substantially reduced subsequent relapse rate [5]. By 1981, in Britain, a combination of rifampicin and isoniazid was the recommended treatment [6]. DOTS (directly observed, short course therapy) was rolled out worldwide in 1995 [7]. A previous modelling study assumed transmissible MDR-M.tb strains arose 20-60 years before 2013 (1953 1993) [8].

Hence, MDR is assumed to have appeared at detectable levels in a country somewhere between 1970 and 2000 (i.e. 1970 ¡ t_m ¡ 2000). Assuming that the initial uptake of TB treatment was slow [9], we assume a mean appearance in 1985, with a 95% confidence interval that it was between 1970 and 2000. Hence:

$$t_m \sim N(1985, 9) \quad (1)$$

1.3.2 Equation for trend

Given this time, we then assumed that the proportion of new TB cases that were MDR (y) could increase in the form of a quadratic, where t is time.

$$y = bt - ct^2 \quad (2)$$

We would like there to be no MDR-TB before t_m. Hence we use the formulation:

$$y = b(t - t_m) - c(t - t_m)^2 = (t - t_m)(b - c(t - t_m)) \quad (3)$$

This sets the proportion of new TB cases that were MDR to be zero at t_m. In order for the trend to increase from time t_m (when MDR first appeared) and then (potentially) reach the second zero we need the second solution of this equation to occur at $t > t_m > 0$. If t_m were zero, then $y = 0$ at $t = \frac{b}{c}$. Otherwise, for the second zero to occur after t_m requires:

$$t = \frac{b + ct_m}{c} > t_m => b > 0 \quad (4)$$

What we would like to assume is that the second zero occurs at a time after 2014 as it is unlikely that any trend in the proportion of new TB cases that were MDR increases from zero at time t_m, peaks and declines within the 1970-2014 period. If we make the

assumption that $c = \frac{rb}{t_m}$ we can calculate limits on the newly introduced scalar parameter r that prevent this occurring. If we assume the second zero of MDR-TB is after 2014 then

$$\frac{b + ct_m}{c} > 2014 \quad c = \frac{rb}{t_m} \tag{5}$$

$$\frac{b + \frac{rb}{t_m}t_m}{\frac{rb}{t_m}} > 2014 \tag{6}$$

$$=> \frac{t_m(1+r)}{r} > 2014 \tag{7}$$

$$=> r < \frac{t_m}{2014 - t_m} \tag{8}$$

If we assume that the very earliest that t_m can be is 1960, then in order for the second zero to occur after 2014, r must be less than 36 (substitute 1960 for t_m into (8)). Here r is the scalar parameter for calculation of c from b and t_m. This assumes in general that from MDR-TB appearance to disappearance cannot take less than 55 years. For example, if $r = 35$, and MDR-TB appears in 1985 ($t_m = 1985$), then the second zero is predicted to occur in 2041.

Assuming a uniform distribution for r is likely to lead to many declining trends in MDR-TB, when we know that few countries have evidence for this [10]. Instead a normal distribution with a mean of 5 and standard deviation of 15 gives a 95% range of values between -20 and 30.

1.3.3 Rate of increase

The rate of increase is dependent on both b and c. Previous modelling work has suggested that for those with a statistically significant linear trend in estimated per capita rates of MDR-TB among new notified TB cases the range of annual % change is -15% to 21% [10]. Negative trends arise when $c < 0$, by allowing $r < 0$.

We set the prior distribution for the parameter b to be lognormal (as we assumed, for the second zero to occur after t_m, that it must be always positive) with a mean of -5.5 and standard deviation of 0.7. To reach this distribution, we assumed an upper limit of 1% for b. This prior was then tuned, within the multivariate analysis below, in order to give lower increases (around 0.1%) for the full range of WHO data trends to be captured.

1.3.4 Proposed priors

We can use this to generate priors for three parameters:

$$t_m \sim N(1985, 9)$$
$$b \sim lognormal(-5.5, 0.7)$$
$$r \sim N(5, 15)T[, 36]$$

Here r has a truncated normal prior and is the dummy variable which allows us to calculate c from b and t_m (the time when MDR first appears in a country):

$$c = \frac{rb}{t_m} \tag{9}$$

These are summarised in Table 1.

Parameter	Distribution	Prior	Notes
Time of MDR appearance (t_m)	Normal	$N(1985, 9)$	95% range between 1970 and 2000
Coefficient of linear term (b)	Log-Normal	$Lognormal(-5.5, 0.7)$	Skewed to low rates of increase and always positive
Scalar for linking b and c (r)	Truncated Normal	$N(5, 15)$, truncated to be below 36	Capped at 36 to prevent decrease of MDR to zero before 2014

Table 1: Prior details for our model for trend: $y = bt - ct^2$. Here y is the proportion of new TB cases with MDR, t is time, b is the coefficient of the rate of linear increase and c is the coefficient of the rate of quadratic change. The scalar term (r) is used to calculate the coefficient of the quadratic term $c = rb/t$.

1.3.5 Effect of each parameter on trend

A uni-variate analysis of the output by parameter is shown in Figure 2, with a two-way variation analysis shown in Figure 3a&b. Also shown here is the available data from the WHO DRS (for 138 countries, Figure 3c). These prior outputs are only examples for one origin (t_m) time point for MDR (Figure 3a is 1975, Figure 3b is 1985), hence we would not expect perfect overlap of the output from the priors and the WHO DRS data. However, a comparison is useful as the overall levels can be seen to be similar.

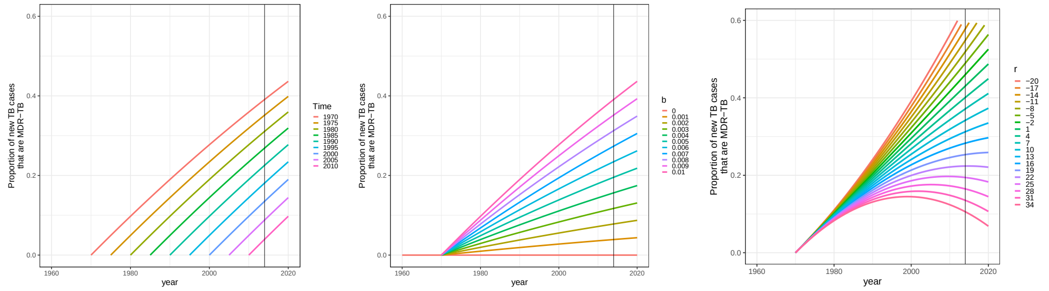

(a) t_m **(b)** b **(c)** r

Figure 2: Univariate analysis of changes in trend in proportion of new TB cases that are MDR. (a) Variation in t_m with $b = 0.01$ and $r = 5$. (b) Variation in b with $t_m = 1970$ and $r = 5$. (c) Variation in r with $t_m = 1970$ and $b = 0.01$. Note that here the limit of $r < 36$ prevents too steep a decline in MDR-TB. The dashed vertical line is 2014, the year we are estimating MDR-LTBI for.

1.3.6 Model fitting details

We used 2 separate Markov Chain Monte Carlo runs of 20,000 iterations with the first 50% discarded as ”burn-in” and the remainder thinned by a factor of 10. We took the samples as the last 200 from the first chain.

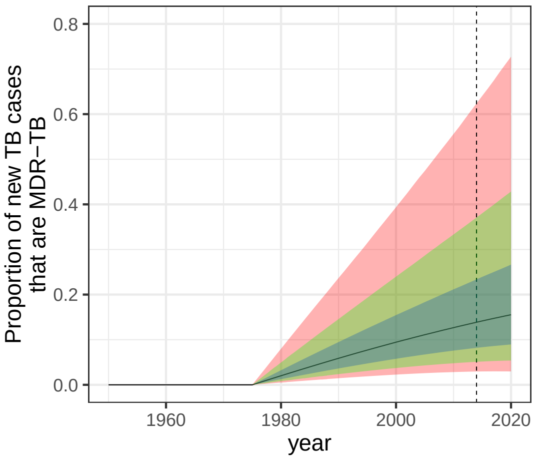

(a) Prediction from priors with t_m = 1975

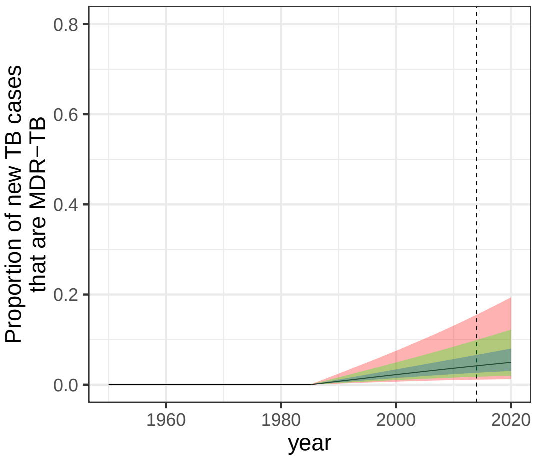

(b) Prediction from priors with t_m = 1985

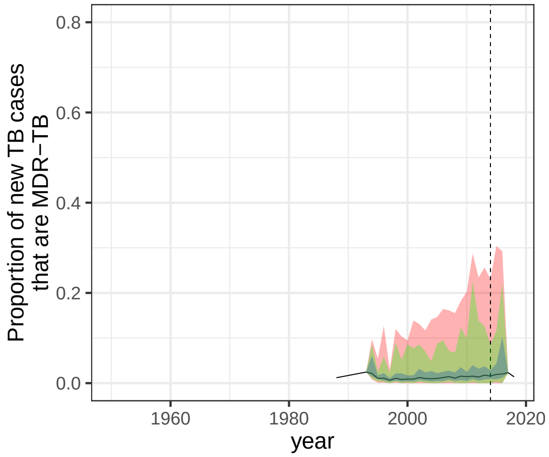

(c) WHO data

Figure 3: Prior output vs. data. The black line is the median value in each year. Shaded areas show 95% (red), 80% (light green) and 50% (dark green) ranges for (a) trends varying prior values for b and r with $t_m = 1975$, (b) trends varying prior values for b and r with $t_m = 1985$ and (c) WHO DRS data in proportion of new cases of TB with MDR. The dashed vertical line is 2014, the year we are estimating MDR-LTBI for.

1.4 Sensitivity analysis 3: trend analysis

We also considered more complex curves for some settings in which we deemed there to be sufficient information and trends in the data that suggested more complex dynamics in MDR-TB ARI. In particular, several settings had what could be deemed ”peak and crash” dynamics: major changes in MDR-TB treatment practices might be expected to produce an asymmetric trend in ARI, with a peak followed by a (rapid) decline and then potentially continued growth but at a reduced rate.

1.4.1 Countries included in sensitivity analysis 3

To reduce the chance of overfitting we followed the below algorithm to perform the sensitivity analysis only in settings that contributed significantly to global MDR-TB burden in 2016 and had what we deemed to be ”sufficient” data.

1. *Countries with sufficient data* The first step was to determine which countries had sufficient data to which a more complex curve could be fitted. We set this to be at least five data points, which leaves 49 countries from the 138.

2. *Peak before 2010* The second step was to remove those with no discernible ”peak and crash” dynamics. To do this we set that a peak level in the data had to occur before 2010 (to allow for at least 5 years for the post peak trend). We calculated the moving average level (over 3 years) from the data to smooth out outliers. This left a subset of 23 countries (Figure 4).

3. *Sufficient contribution to MDR levels* Of these 23, only China and India are in the top 30 MDR burden countries. Indeed, these two countries are the only ones that contribute more than 0.1% of the total incident MDR-TB burden in 2016. Of our estimates of the number of people with MDR-LTBI in 2014, each of these 23 countries contributed less than 0.1% of the burden apart from China (30%), India (21%) and the USA (0.17%).

Following this algorithm we performed the more flexible model fitting for just these three countries: China, India and the USA.

1.4.2 Complex curve fitting in sensitivity analysis 3

We used the ”smooth.spline” function in *R* to fit smoothed splines to the WHO data for the three final countries. This is a highly flexible model that we fit to the WHO data on the proportion of new TB cases that were MDR with an additional zero data point at 1970. As

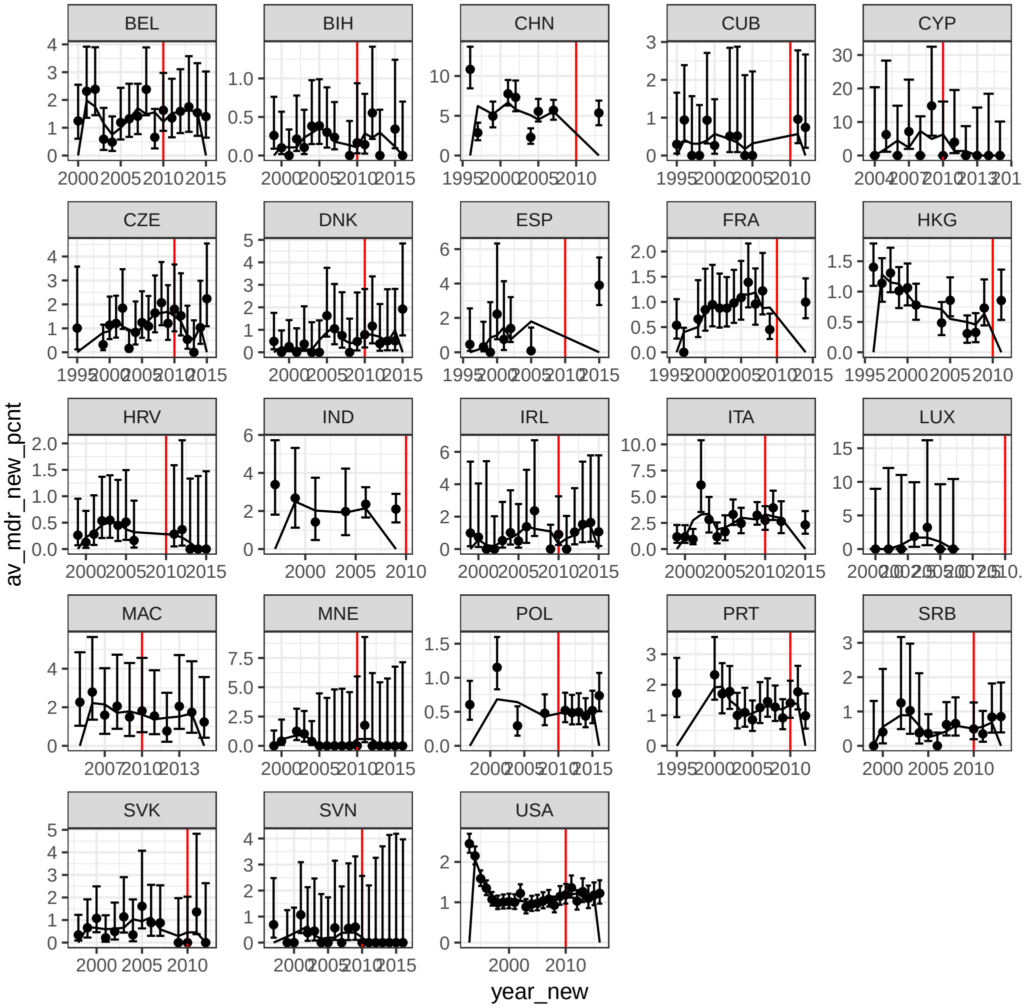

Figure 4: The 23 countries with sufficient data and potential ”peak and crash” dynamics. The red line at 2010 was taken as the cut-off before which a peak should have occured to be included in this sensitivity analysis. The line is the 3 year moving average.

this function fits exactly to the data given, we took 200 samples of ”data”: for each time point with data, we sampled from a normal distribution with mean equal to the average data point and a standard deviation calculated assuming the upper and lower limits of the data represented a 95% confidence interval. These 200 samples then multiplied the ARI from the previous study to give DS- and MDR-ARI over time as in the main analysis.

As the number of data points varied for each country, we had to vary the internal smooth parameter for the spline function (*lambda*). For the USA, China and India we used *lambda* values of 5×10^{-5}, 5×10^{-5} and 1×10^{-3} respectively. These values were chosen to minimise negative predictions and to prevent overfitting.

1.5 Cohort model

1.5.1 Combining the data

200 ARI with *M.tb* trends from Houben & Dodd [1] were multiplied by 200 fitted proportion of new TB cases with MDR trends. The latter were 200 posterior samples. The 95% uncertainty range generated across these 200 trends was reported.

1.5.2 Model construction

The model function (*cohort_ltbi_mdr*, built in R), requires two inputs: the ARI for DS-TB and MDR-TB over time and the population size in 2014. Two matrices are constructed (”now” & ”last”), which track the following infections states, for each age group (row), for a certain year (column):

1. the total proportion infected with DS-TB at the start of the year (p_s)
2. the total proportion infected with MDR-TB at the start of the year (p_r)
3. the new proportion infected with DS-TB during the year (p_{ns})
4. the new proportion infected with MDR-TB during the year(p_{nr})
5. the proportion with DS-TB reinfected with MDR-TB during the year (p_{resr})
6. the proportion with MDR-TB reinfected with DS-TB during the year (p_{rers})
7. the proportion with DS-TB reinfected with DS-TB during the year (p_{ress})
8. the proportion with MDR-TB reinfected with MDR-TB during the year (p_{rerr})

The simulation runs with a time step of a year. The two matrices are: ”now” which tracks this year’s population and ”last”, which stores the population state from the previous year. Both matrices are stored to give the full time series. One hundred, 1-year age groups are included. The algorithm is as follows:

1. Initial conditions: Initialize proportion of the population infected with DS-TB (1934 so no MDR-TB), see section below
2. Aging: At the start of the year the proportions in each of the infection states for age groups 2-100 in the ”now” matrix are set to equal the infection state proportions in the 1-99 ”last” matrix (latest state of the population).

3. Current total infection: The total proportion infected with DS-TB in "now" matrix is the sum of the proportion previously infected in "last" plus the new and re-infected proportions where the infecting strain changes ($p_s = p_s + p_{ns} + p_{rers}$)
4. The same calculations is performed for the proportion infected with MDR-TB ($p_r = p_r + p_{nr} + p_{resr}$)
5. The new and reinfected proportions in the "now" matrix are set to zero
6. The ari for DS-/MDR-TB for this year are selected from the input matrix
7. New infections: The proportion of the uninfected population that is newly infected with DS-TB in this year is calculated ($p_{ns} = ari_{DS} \times (1 - p_s - p_r)$)
8. The proportion of the uninfected population that is newly infected with MDR-TB in this year is calculated ($p_{nr} = ari_{DR} \times (1 - p_s - p_r - p_{ns})$) taking into account that some have newly been infected with DS-TB
9. Reinfections: The proportion of reinfections is calculated for those with DS-TB that get successfully re-infected with DR-TB ($p_{resr} = ari_{DR} \times \alpha p_s$). Here α is the level of protection from [11] (randomly sampled from beta distribution with mean 0.79 (and range 0.7-0.86) as in [1]).
10. The proportion of reinfections is calculated for those with MDR-TB that get successfully re-infected with DS-TB ($p_{rers} = ari_{DS} \times \alpha p_r$)
11. The proportion of reinfections that do not change the infecting strain (p_{ress} and p_{rerr}) are calculated for estimating the levels of recent infection.
12. Set "last" matrix to be this "now" matrix and store
13. Repeat 2-12 for all years (1934 - 2014)
14. Multiply the proportions in 2014 by the UN population size in each age group (1-100)

1.5.3 Initial conditions

The initial conditions are calculated assuming a constant ARI pre-1934 which is the ARI value in 1934 (ari_{1934}). There is assumed to be no MDR-TB pre-1960. The initial proportion infected with DS-TB in each age group *i* is

$$P_i = 1 - exp(-i \times ari_{1934}) \tag{10}$$

1.5.4 Key assumptions

The key assumptions that this cohort model makes are that

- LTBI is with the last strain to cause infection. This simplifies the analysis: we do not consider the impact of mixed infections nor the impact of re-infection on reactivation of previous or newly infecting strains.
- Annual risk of infection is homogeneous across all age groups.
- Infection with susceptible strains happens ”first” in a year. This shouldn’t affect results.

1.6 Metric for MDR-LTBI data coverage

Using the cohort model, we can track when infections with MDR-*M.tb* occurred and hence calculate how much MDR-LTBI was acquired in each 5 year time period in the past.

To do this, we took the prevalence of LTBI (DS or MDR) in each of 100 age groups in 2014. We then asked from which year was this LTBI acquired. For example, those aged 20 in 2014 were age 1 in 1995. By looking at the prevalence of LTBI in one year olds in 1995 and how this changed to two year olds in 1996, we can ask what proportion of the LTBI prevalence in 2014 was acquired at each time point. As the LTBI prevalence can decrease (especially for DS-TB), we calculated the sum of all the positive cumulative increases in prevalence of the lifetime of each age group and divided by their total to give percentage contributions for each year. This makes the assumption that any decrease in prevalence removes LTBI acquired equally across all previous years. Only the cumulative change is considered (i.e. the proportion that was due to assumed DS infection prior to 1934 is removed).

As the prevalence of LTBI varies by age, and the age groups are different sizes, these need to be taken into account when estimating when contributed most to LTBI burden in 2014. Hence, the population size in 2014 is used to convert the prevalence of LTBI at each age group into the actual numbers with LTBI in 2014. The proportions of the total population with LTBI in 2014 that was infected in each previous 5 yr time unit was then derived.

These proportions were multiplied by a 0 or 1 dependent on the availability of WHO data in that 5 yr time unit. This gives a metric which is 1 when all time periods that contribute to MDR-LTBI burden have supporting data.

1.7 Code

The code for the cohort model and calculations of trend, with some data and country level results, can be found at:
https://github.com/gwenknight/MDR-LTBI-estimates

2 Additional Results

2.1 Top 10 countries by number with MDR-LTBI

The 10 countries with the higher number of people with MDR-LTBI is shown in Figure 5. Note that although China and India dominate in terms of numbers, they have a low prevalence of MDR-LTBI.

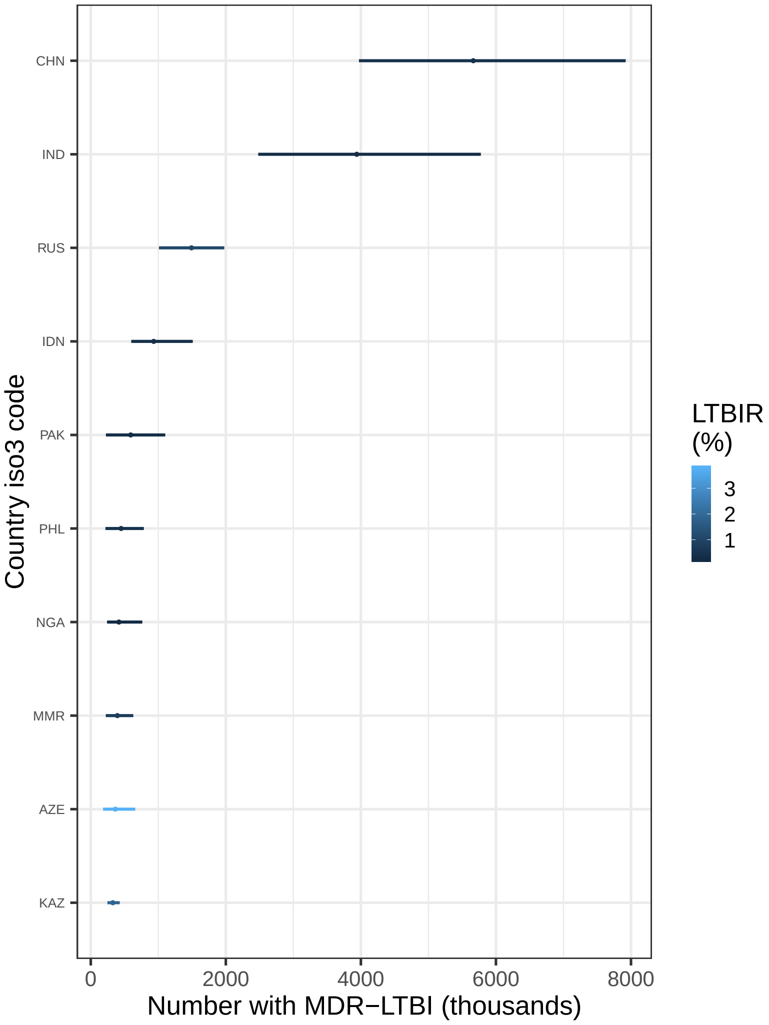

Figure 5: The top 10 countries by MDR-LTBI burden (country's labelled by their iso3 codes). The prevalence of MDR-LTBI is given in the colour scale.

2.2 Model fits for each country

The 200 model fits for each country are given in Figure 6. Here, both the linear and quadratic trends can be seen, for example in Bulgaria (”BGR”) and Chile (”CHL”). Our assumptions about a not-too drastic rate of MDR increase means that we do not fit some of the higher, earlier data points (e.g. for Iran ”IRN” and Morocco ”MAR”).

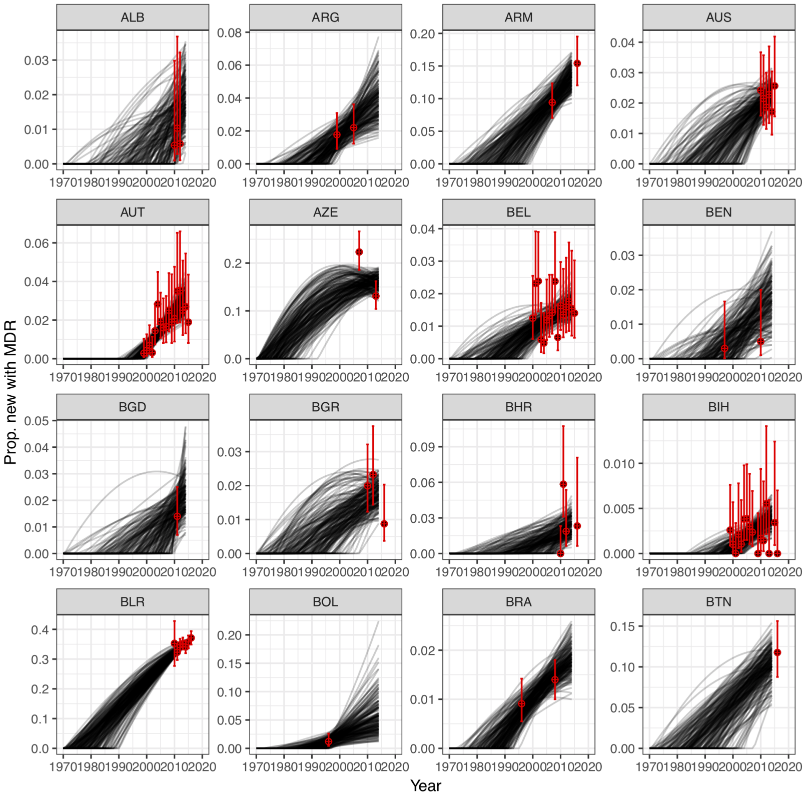

(a) Countries ALB - BTN

Figure 6: Model fits (black lines) to WHO data for proportion of new TB that is MDR (red dots and errorbars). Country names are given by their iso3 codes.

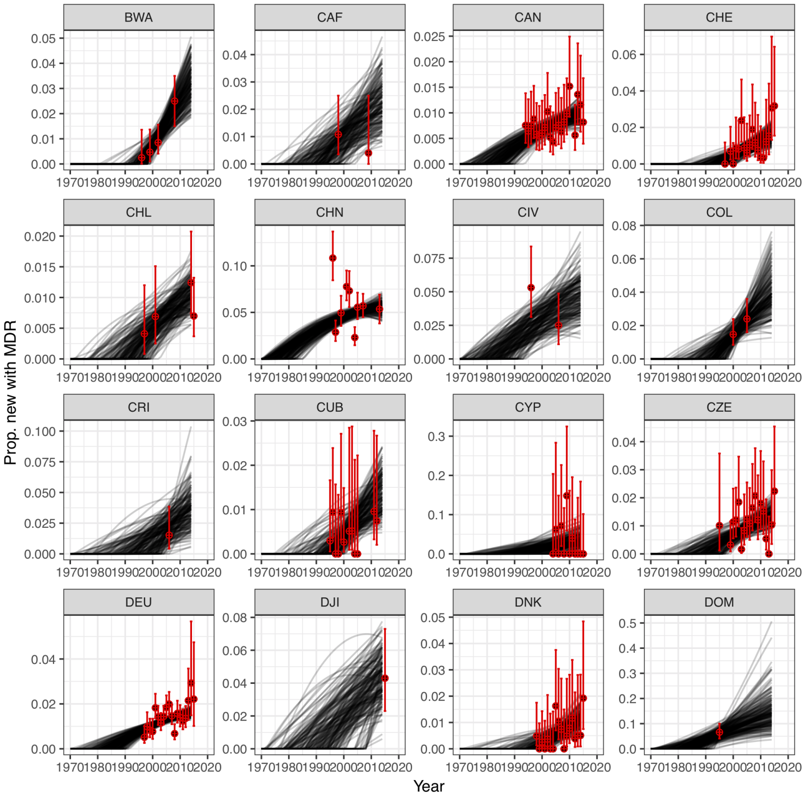

(b) Countries BWA-DOM

Figure 6: Model fits (black lines) to WHO data for proportion of new TB that is MDR (red dots and errorbars). Country names are given by their iso3 codes.

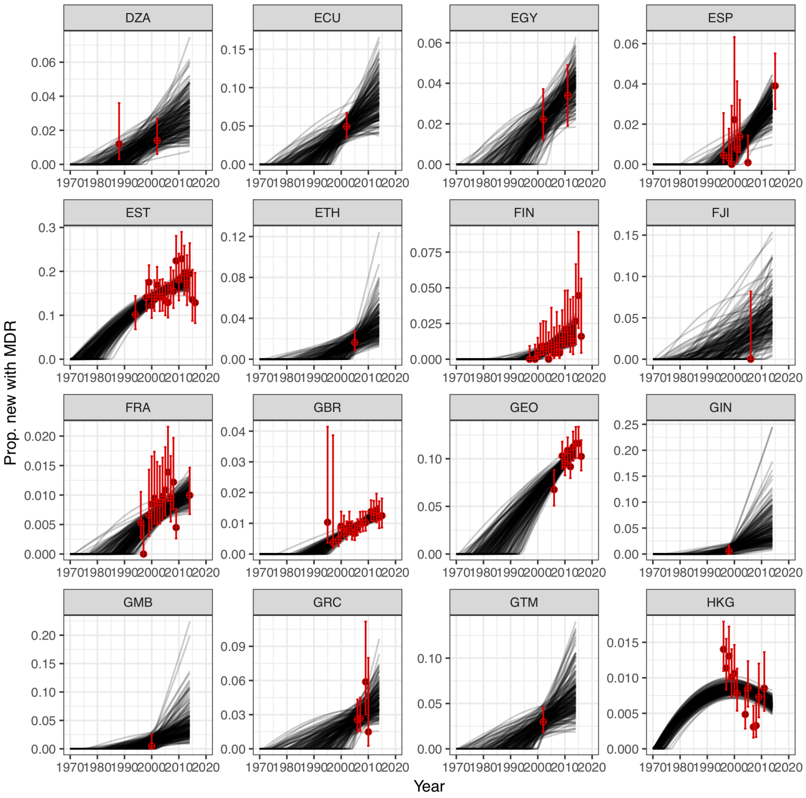

(c) Countries DZA - HKG

Figure 6: Model fits (black lines) to WHO data for proportion of new TB that is MDR (red dots and errorbars). Country names are given by their iso3 codes.

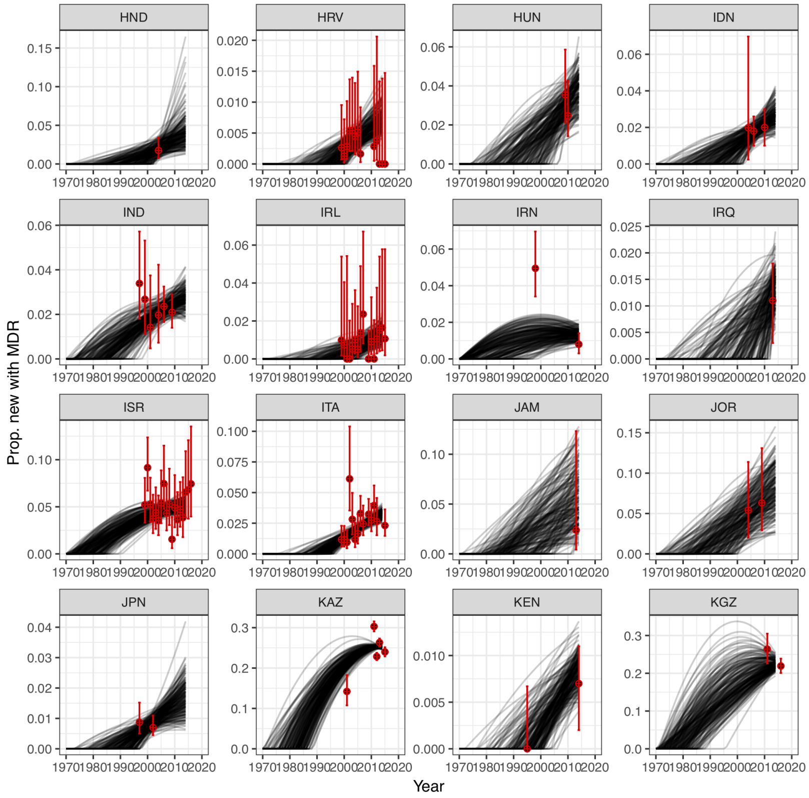

(d) Countries HND - KGZ

Figure 6: Model fits (black lines) to WHO data for proportion of new TB that is MDR (red dots and errorbars). Country names are given by their iso3 codes.

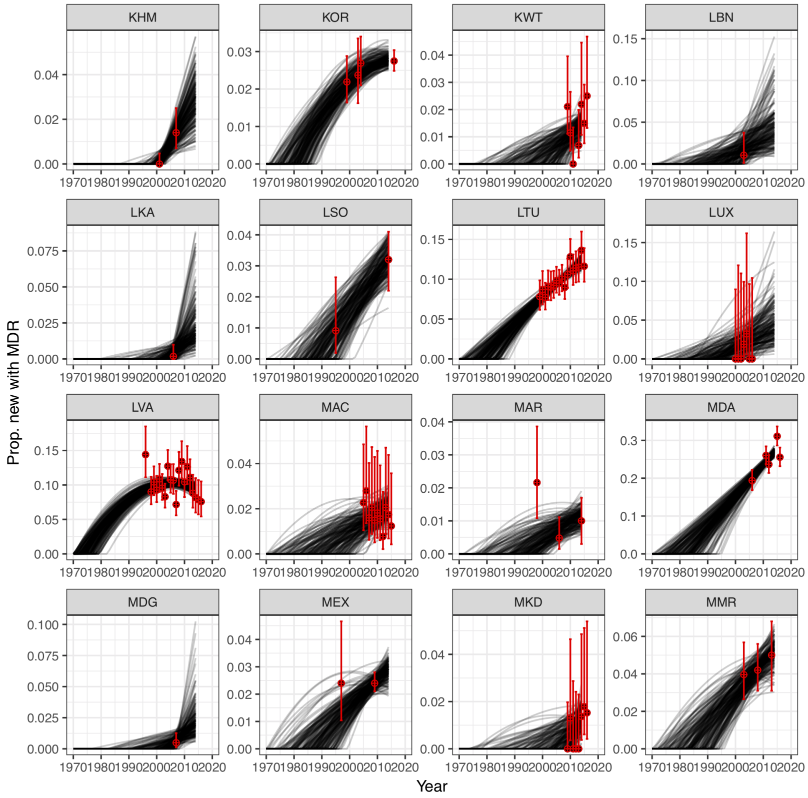

(e) Countries KHM - MMR

Figure 6: Model fits (black lines) to WHO data for proportion of new TB that is MDR (red dots and errorbars). Country names are given by their iso3 codes.

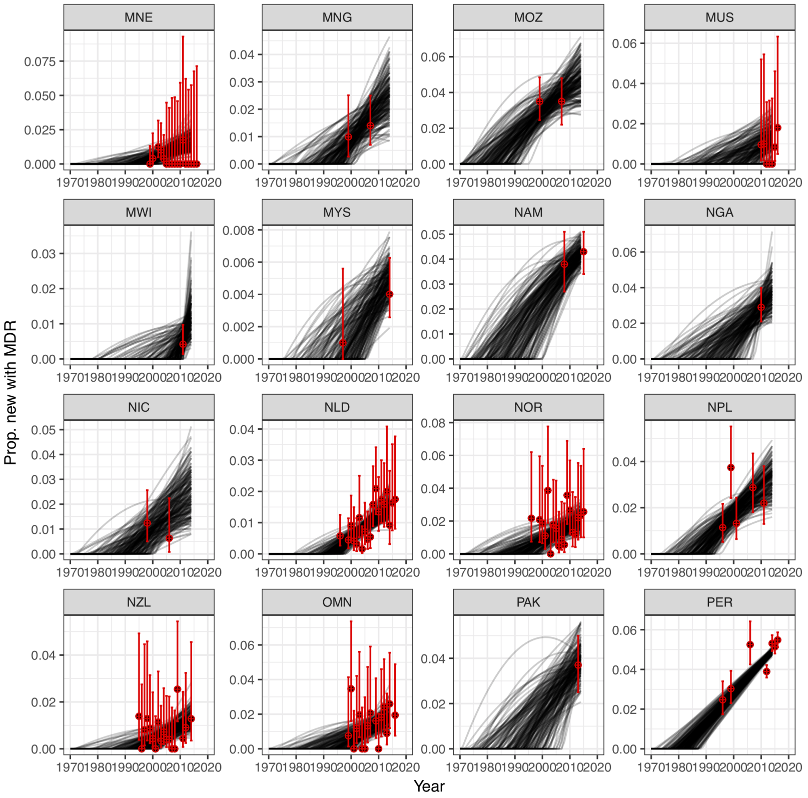

(f) Countries MNE - PER

Figure 6: Model fits (black lines) to WHO data for proportion of new TB that is MDR (red dots and errorbars). Country names are given by their iso3 codes.

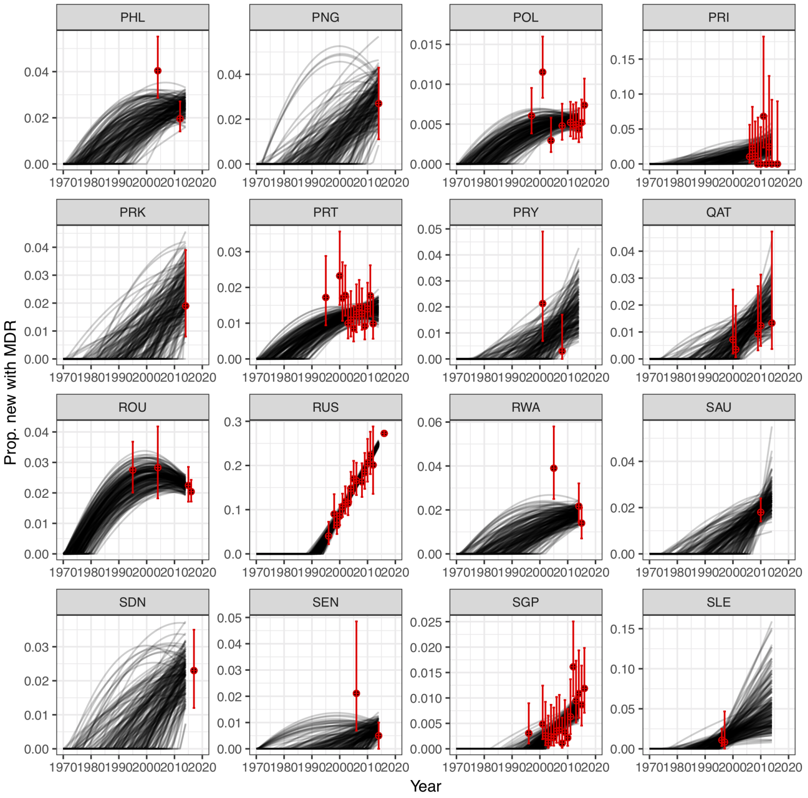

(g) Countries PHL - SLE

Figure 6: Model fits (black lines) to WHO data for proportion of new TB that is MDR (red dots and errorbars). Country names are given by their iso3 codes.

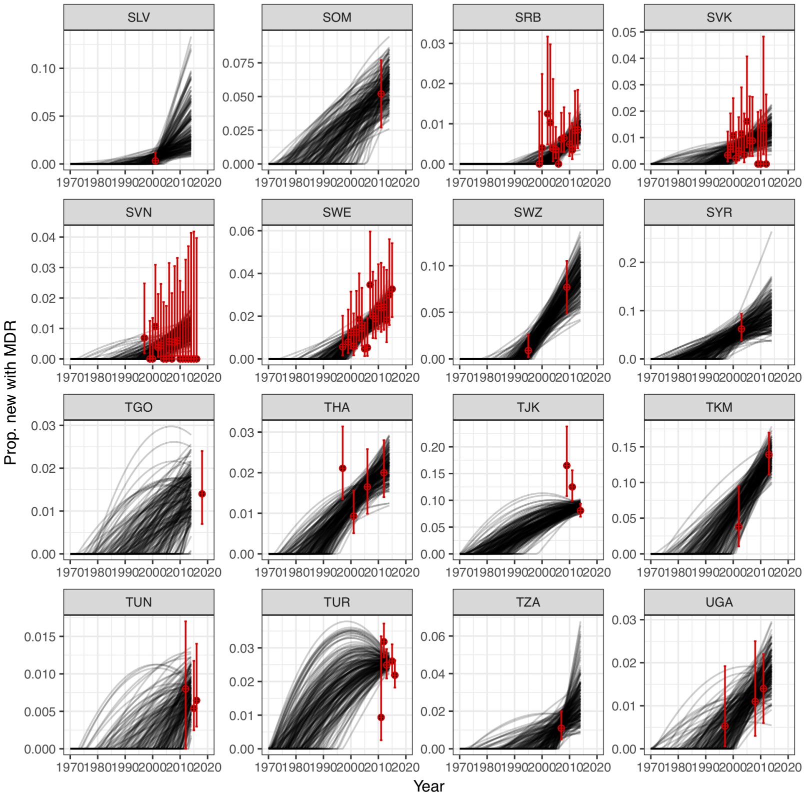

(h) Countries SLV - UGA

Figure 6: Model fits (black lines) to WHO data for proportion of new TB that is MDR (red dots and errorbars). Country names are given by their iso3 codes.

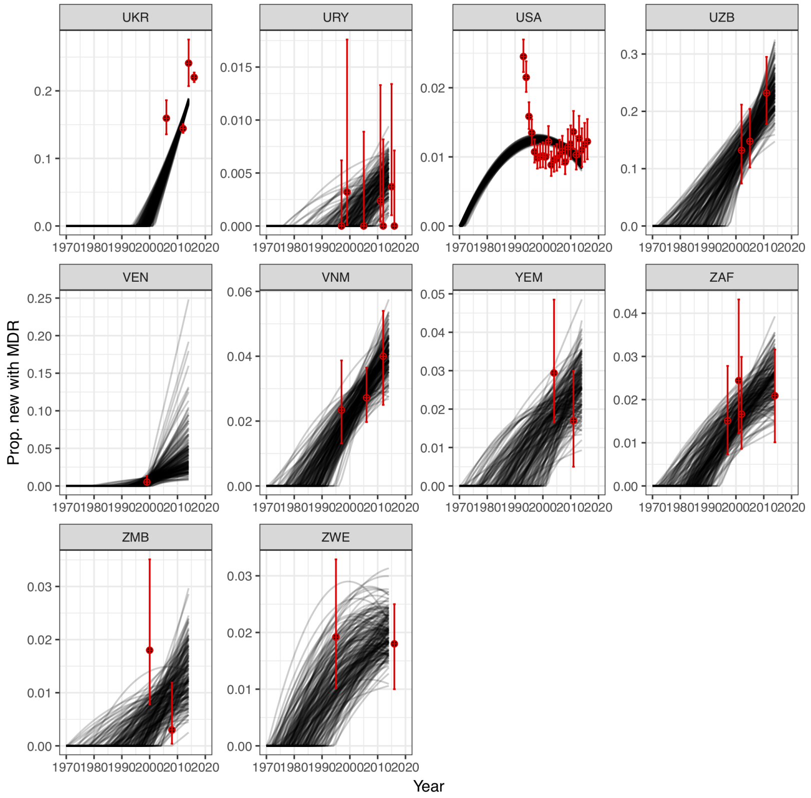

(i) Countries UKR - ZWE

Figure 6: Model fits (black lines) to WHO data for proportion of new TB that is MDR (red dots and errorbars). Country names are given by their iso3 codes.

2.3 Proportion infected by age

The proportion with DS-LTBI or MDR-LTBI is given in Figure 7.

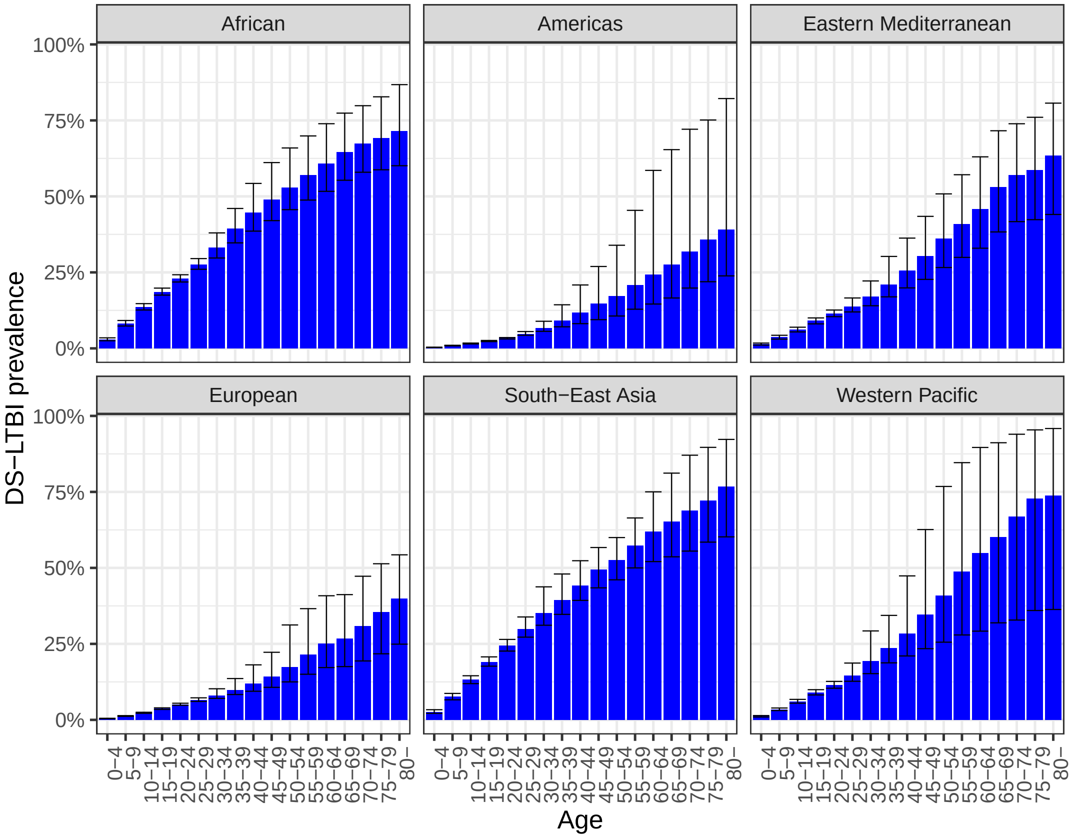

Figure 7: The percentage of the population for each of the six WHO regions carrying DS-LTBI. The MDR-LTBI levels are shown in the main text. Error bars indicate 95% uncertainty interval.

2.4 Metric for MDR-LTBI data availability

Our metric for MDR-LTBI data availability is 1 when data is available in all 5-year time periods in which some MDR-LTBI in 2014 are acquired. A value of 0.5 suggests that 50% of the MDR-LTBI burden comes from time periods with data. Many countries have values of this metric above 0.5 (Figure 8). However, the variation with trend is high (Figure 9) and some countries have few data and so always low MDR-LTBI data coverage.

For the top 30 countries (Figure 10) most had values below 0.5. An example of the contributing 5-year time period levels for Botswana is given in Figure 11.

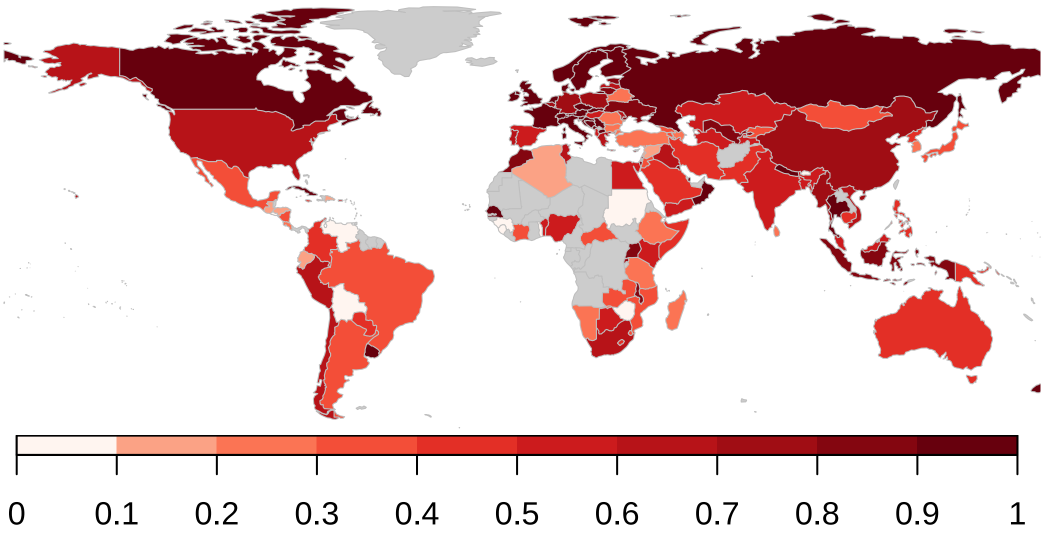

Figure 8: MDR-LTBI data availability metric median values for each of the 138 countries used in this analysis. Countries with no data are shown in grey.

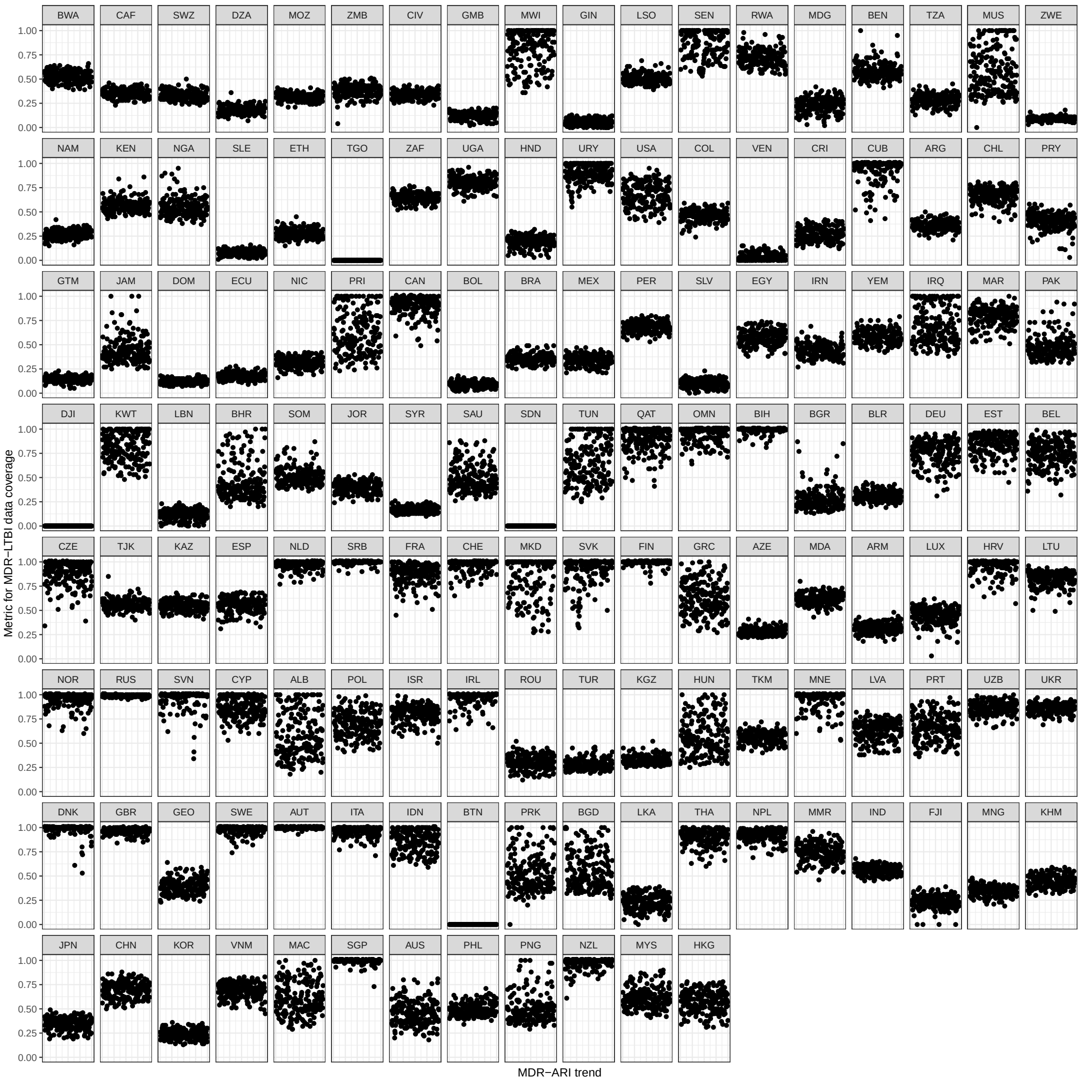

Figure 9: MDR-LTBI data availability metric for each country (grey bar at top indicates iso3 code) and each of the 200 MDR-ARI trends (each individual black dot).

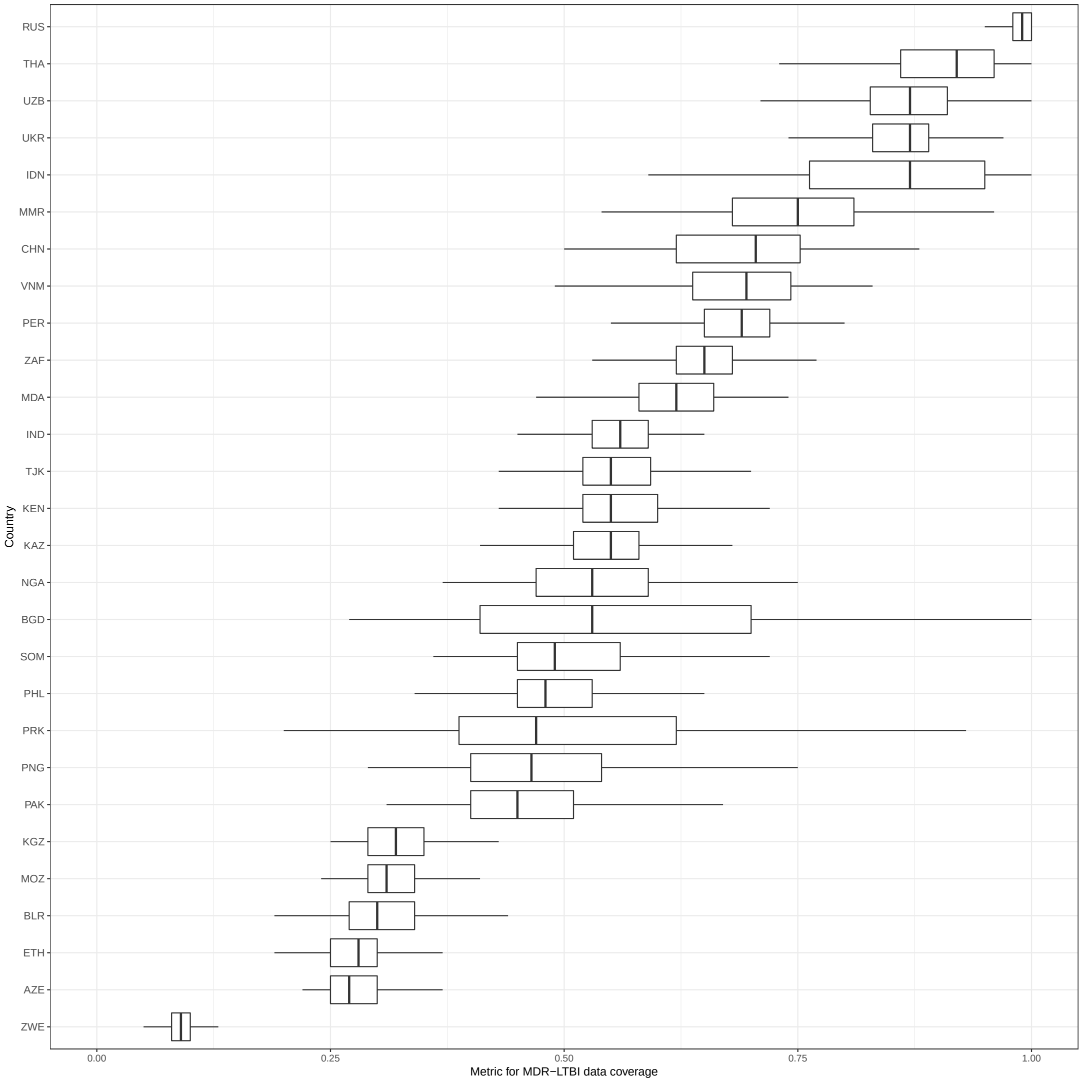

Figure 10: Boxplot of metric for MDR data availability ranked by the median of this metric for the top 30 high MDR burden countries. Countries are labelled by their iso3 codes.

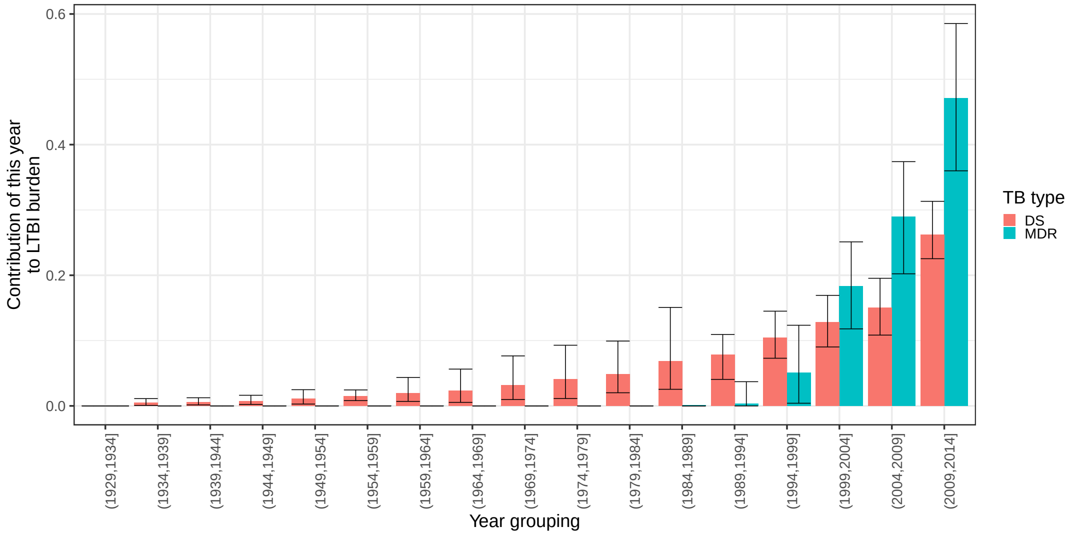

Figure 11: Contribution of each 5-year time period to DS- (red) and MDR- (blue) LTBI burden for Botswana. Error bars are 95% uncertainty intervals over the 200 model fits.

2.5 Sensitivity analysis 1

Reducing the protective effect of MDR-LTBI to reinfection results in output that is very similar to the main results (Table 2).

**WHO Region**	**DS-LTBI prevalence (%)**	**MDR-LTBI prevalence (%)**	**LTBI that is MDR (%)**	**LTBI that is MDR in <15 year olds (%)**
African	22.1 [20.1-25.5]	0.22 [0.18-0.28]	1.0 [0.8-1.3]	2.3 [1.9-2.6]
Americas	10.6 [7.3-19.0]	0.05 [0.04-0.06]	0.5 [0.3-0.8]	3.3 [2.8-4.1]
South-East Asia	30.7 [27.7-34.5]	0.29 [0.22-0.39]	1.0 [0.7-1.3]	2.2 [1.9-2.6]
Eastern Mediterranean	16.4 [13.5-20.9]	0.14 [0.08-0.23]	0.9 [0.5-1.5]	2.8 [1.9-3.7]
Western Pacific	26.8 [17.8-39.2]	0.35 [0.26-0.47]	1.3 [0.7-2.1]	3.7 [3.3-4.1]
European	13.5 [9.9-19.8]	0.37 [0.31-0.43]	2.7 [1.6-3.8]	14.0 [13.1-15.1]
GLOBAL	22.9 [20.1-26.1]	0.27 [0.23-0.30]	1.2 [0.9-1.4]	2.8 [2.6-3.1]

Table 2: Proportion of population by WHO region infected with *Mycobacterium tuberculosis*, 2014. Brackets indicate 95% uncertainty interval.

2.6 Individual country estimates

Individual country estimates in an Excel spreadsheet can be found at:
https://github.com/gwenknight/MDR-LTBI-estimates

2.7 Map of MDR-LTBI prevalence in 2035 and 2050

Assuming no ongoing transmission after 2014, the mapped estimated population prevalence of MDR-LTBI (%) in 2035 and 2050 can be seen in Figures 12&13 respectively.

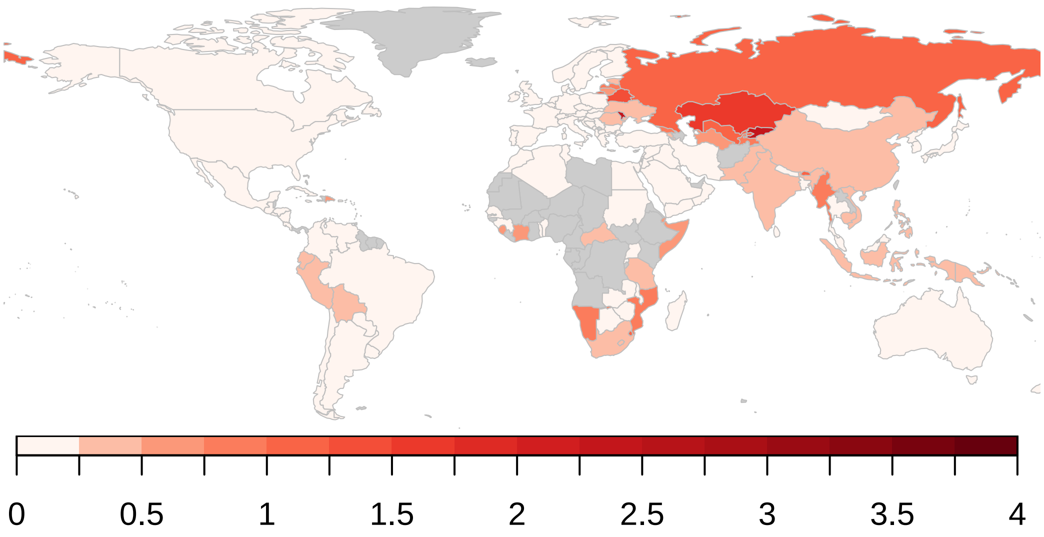

Figure 12: Estimated population prevalence of MDR-LTBI (%) in 2035 with no ongoing transmission after 2014. Countries with no data shown in grey.

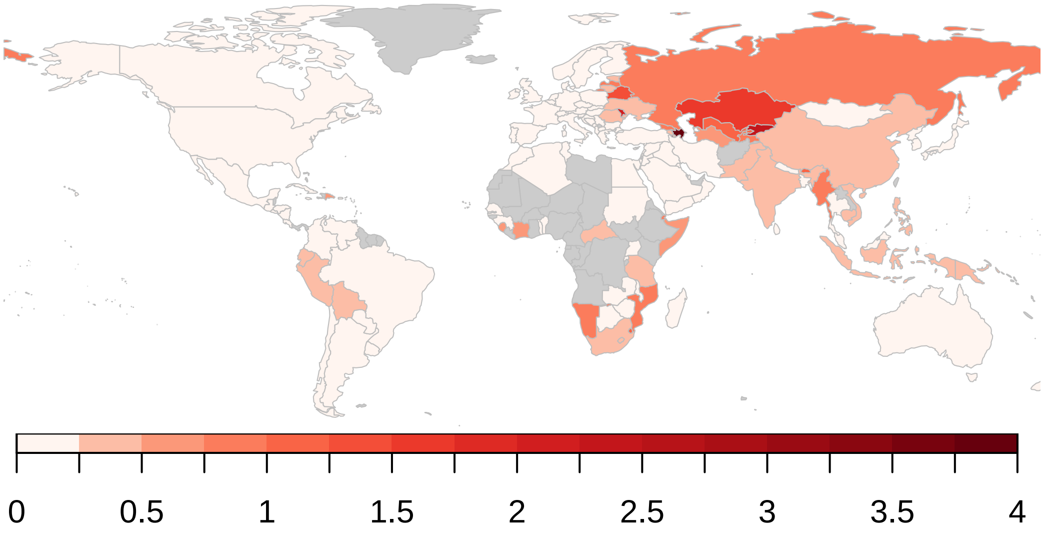

Figure 13: Estimated population prevalence of MDR-LTBI (%) in 2050 with no ongoing transmission after 2014. Countries with no data shown in grey.

2.8 Sensitivity analysis 3: trend analysis

2.8.1 Spline curve fit

The resulting spline fit to the proportion of new TB cases that are MDR is shown in Figure 14.

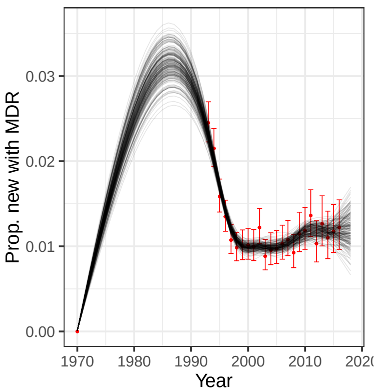

(a) USA

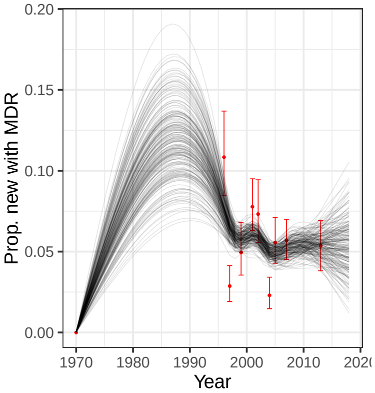

(b) China

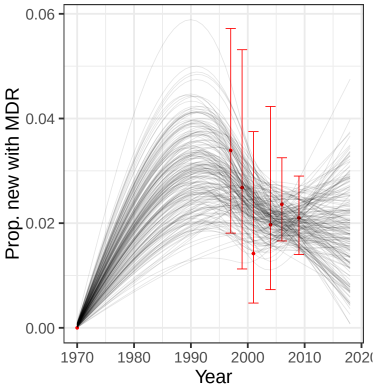

(c) India

Figure 14: Sensitivity analysis 3 on model fits (smooth splines, black lines) to WHO data for proportion of new TB that is MDR (red dots and errorbars).

2.8.2 Estimated levels

The new results are shown in Table 3. Note that for the three countries explored in this sensitivity analysis: the USA is in the Americas, India is in the South-East Asia and China

is in the Western Pacific WHO region. As expected, the values for the African, Eastern Mediterranean and European regions do not change.

At the country level, the prevalence of MDR-LTBI increases from 0.41 [95% UI 0.29 - 0.58] to 1.12 [95% UI 0.76 - 1.94] in China, from 0.30 [95% UI 0.19 - 0.45] to 0.50 [95% UI 0.33 - 0.77] in India and from 0.01 [95% UI 0.01 - 0.03] to 0.02 [95% UI 0.01 - 0.08] in the USA. This results in higher percentages of LTBI that are MDR: 4.5 [95% UI 2.5-7.0], 1.9 [95% UI 1.3-2.6], 0.7 [95% UI 0.2-1.7] in China, India and the USA respectively up from 1.6 [95% UI 0.8-2.9], 1.1 [95% UI 0.7-1.7] and 0.4 [95% UI 0.1-1.0].

At the global level, due to the large contribution of India and China, the percentage of LTBI that is MDR increases from 1.2 [95% UI 1.0 - 1.4] to 2.0 [95%UI 1.6 - 2.6], whilst the percentage of LTBI that is MDR in children remains basically unchanged (Table 3). This latter result highlights that the change in trend mostly affects the dynamics of MDR-ARI in the 1980-2000s when there are few data and in this analysis, the potential for rapid and large increases in the MDR-ARI levels. Children (those ¡ 15 years old), would not have been alive at this time and so their MDR-LTBI levels are unaffected.

Despite having relatively similar population sizes (1.3 billion), the contribution of China to the change in the overall numbers with MDR-LTBI was much greater than India: 77% of the increase in the number of MDR-LTBI infected individuals was due to China vs. 20% and 3% for India and the USA respectively. This is driven by the higher proportion of new TB cases having MDR-TB in China (Figure 14).

These estimates are higher than those reported in the main analysis for two main reasons: firstly here we assumed MDR-TB appeared in a country in 1970 (earlier than could happen in the main analysis). Secondly, we allowed for a peak in the proportion of new TB cases that are MDR to occur in the time period when there was little to no supporting data.

**WHO Region**	**DS-LTBI prevalence (%)**	**MDR-LTBI prevalence (%)**	**LTBI that is MDR (%)**	**LTBI that is MDR in <15 year olds (%)**
African	22.1 [20.1-25.5]	0.23 [0.19-0.29]	1.0 [0.8-1.3]	2.3 [1.9-2.7]
Americas	10.6 [7.3-19.0]	0.06 [0.05-0.08]	0.5 [0.3-0.8]	3.3 [2.8-4.1]
South-East Asia	30.5 [27.6-33.9]	0.44 [0.33-0.61]	1.4 [1.1-1.9]	2.1 [1.7-2.5]
Eastern Mediterranean	16.4 [13.5-20.9]	0.14 [0.08-0.24]	0.9 [0.5-1.5]	2.9 [1.9-3.8]
Western Pacific	25.9 [17.3-38.7]	0.89 [0.61-1.49]	3.4 [2.0-5.0]	3.8 [3.3-4.2]
European	13.5 [9.9-19.8]	0.38 [0.32-0.44]	2.8 [1.6-3.9]	14.1 [13.1-15.2]
GLOBAL	22.6 [19.9-25.9]	0.46 [0.38-0.62]	2.0 [1.6-2.6]	2.8 [2.5-3.0]

Table 3: Proportion of population infected with *Mycobacterium tuberculosis* of differing drug resistance type, by WHO region, in 2014 within sensitivity analysis 3. Brackets indicate 95% uncertainty interval.

References

[1] R. M. Houben, P. J. Dodd, The global burden of latent tuberculosis infection: a re-estimation using mathematical modelling, PLoS medicine 13 (10) (2016) e1002152.

[2] WHO, Global tuberculosis report (2018).
URL http://www.who.int/tb/publications/global_report/en/

[3] A. Rando-Segura, M. L. Aznar, M. M. Moreno, M. Espasa, E. Sulleiro, C. Bocanegra, E. Gil, A. N. E. Eugnio, C. Escartin, A. Zacarias, J. Vegue, D. Katimba, M. C. Vivas, E. Gabriel, M. C. Marina, J. Mendioroz, M. T. Lpez, T. Pumarola, I. Molina, M. T. Trtola, Drug resistance of mycobacterium tuberculosis complex in a rural setting, angola., Emerging infectious diseases 24 (2018) 569–572. doi:10.3201/eid2403.171562.

[4] M. K. Kaswa, S. Bisuta, G. Kabuya, O. Lunguya, A. Ndongosieme, J. J. Muyembe, A. Van Deun, M. Boelaert, Multi drug resistant tuberculosis in mosango, a rural area in the democratic republic of congo., PloS one 9 (2014) e94618. doi:10.1371/journal.pone.0094618.

[5] W. Fox, G. A. Ellard, D. A. Mitchison, Studies on the treatment of tuberculosis undertaken by the british medical research council tuberculosis units, 1946-1986, with relevant subsequent publications., The international journal of tuberculosis and lung disease : the official journal of the International Union against Tuberculosis and Lung Disease 3 (1999) S231–S279.

[6] Tuberculosis Service of the British Medical Research Council (Singapore), Clinical trial of six-month and four-month regimens of chemotherapy in the treatment of pulmonary tuberculosis: the results up to 30 months, Tubercle 62 (2) (1981) 95–102.

[7] R. Nowak, Who calls for action against tb, Science 267 (5205) (1995) 1763.

[8] E. A. Kendall, M. O. Fofana, D. W. Dowdy, Burden of transmitted multidrug resistance in epidemics of tuberculosis: a transmission modelling analysis, The Lancet Respiratory Medicine 3 (12) (2015) 963–972.

[9] M. C. Raviglione, A. Pio, Evolution of who policies for tuberculosis control, 1948–2001, The Lancet 359 (9308) (2002) 775–780.

[10] T. Cohen, H. E. Jenkins, C. Lu, M. McLaughlin, K. Floyd, M. Zignol, On the spread and control of mdr-tb epidemics: an examination of trends in anti-tuberculosis drug resistance surveillance data, Drug Resistance Updates 17 (4-6) (2014) 105–123.

[11] J. R. Andrews, F. Noubary, R. P. Walensky, R. Cerda, E. Losina, C. R. Horsburgh, Risk of progression to active tuberculosis following reinfection with Mycobacterium tuberculosis, Clin Infect Dis 54 (6) (2012) 784–91.
